# C:N:P stoichiometric variations of herbs and its relationships with soil properties and species relative abundance along the Xiaokai River irrigation in the Yellow River Delta, China

**DOI:** 10.3389/fpls.2023.1130477

**Published:** 2023-01-30

**Authors:** Peipei Jiang, Xiaojun Han, Ziyu Liu, Shoujin Fan, Xuejie Zhang

**Affiliations:** ^1^Dongying Key Laboratory of Salt Tolerance Mechanism and Application of Halophytes, Dongying Institute, Shandong Normal University, Dongying, China; ^2^Key Lab of Plant Stress Research, College of Life Sciences, Shandong Normal University, Ji’nan, China; ^3^Binzhou Yellow River Irrigation Management Service Center, Binzhou, China; ^4^School of Industrial Engineering, Purdue University, West Lafayette, IN, United States

**Keywords:** ecological stoichiometry, salinity stress, seasonal dynamics, spatial variation, intraspecific variation, species relative abundance

## Abstract

**Introduction:**

Soil salinity is known to affect plant performance and nutrient stoichiometry by altering their ecophysiology, and thus playing a crucial role in determining plant distribution patterns and nutrient cycles in salinized ecosystems. However, there was little consensus on the effects of salinity stress on plant C, N, and P stoichiometries. Moreover, determining the relationships between species relative species abundance and plant C, N, and P stoichiometries can help to understand the different adaptive strategies between the common and rare species as well as the community assembly process.

**Methods:**

We determined the plant C, N, P stoichiometries at the community and species levels and the relative abundance of species as well as the corresponding soil properties from five sampling sites along a soil salinity gradient in the Yellow River Delta, China.

**Results and Discussion:**

We found that the C concentration of belowground part increased with soil salinity. Meanwhile, plant community N concentration and C:N ratio tended to decrease with soil salinity, whereas the P concentration, C:P, and N:P ratios exhibited the opposite trends. This indicated that N use efficiency increased, while P use efficiency decreased with soil salinity. Moreover, the decreased N:P ratio indicated that N limitation was gradually aggravated along the soil salinity gradient. The soil C:P ratio and P concentration were the major factors of plant C, N, and P stoichiometries in the early growth stage, whereas the soil pH and P concentration were the major factors of plant C, N, and P stoichiometries in the late growth stage. Compared with that of the rare species, the C:N:P stoichiometry of the most common species was medium. Moreover, the intraspecific variations in the aboveground part N:P ratio and belowground part C concentration showed a significant correlation with species’ relative abundance, which indicated that higher intraspecific trait variation might facilitate greater fitness and survival opportunities in environments with high heterogeneity.

**Conclusion:**

Our results revealed that the plant community C:N:P stoichiometry and its determining soil properties varied with plant tissues as well as sampling seasons, and emphasized the importance of intraspecific variation in determining the functional response of plant communities to salinity stress.

## 1 Introduction

Carbon (C), nitrogen (N), and phosphorus (P) are the three most important macroelements in organisms and play critical roles in adjusting plant energy flow, material circulation, and primary productivity (Aerts and Chapin, 2000; Elser et al., 2000; Elser et al., 2010). Changes in C, N, and P concentrations and their ratios can reflect the response mode of plants in nutrient uptake, utilization, and allocation during various growth stages and conditions (Niklas and Cobb, 2005; Minden and Kleyer, 2014). Therefore, exploring the variation patterns in the C, N, and P stoichiometries of plants along an environmental gradient can help to understand the adaptive strategies of plants to changing environments as well as their potential impact on ecosystem processes (Zhang et al., 2018; Fang et al., 2019; Zhang et al., 2021b).

In recent years, the variation patterns of plant C, N, and P stoichiometry with environmental factors have been researched extensively from regional to global scales (Reich and Oleksyn, 2004; Han et al., 2005; Thompson et al., 2010; Gargallo-Garriga et al., 2015; Huang et al., 2019; Luo et al., 2021). For example, leaf N and P concentrations increased and the N:P ratio decreased with increasing latitude and decreasing mean annual temperature (MAT) at the global level (Reich and Oleksyn, 2004). Similar results were found at the regional scale in China, while the difference was that the N:P ratio was weakly associated with latitude and MAT (Han et al., 2005; Ren et al., 2007; Fang et al., 2019). In addition, leaf C concentration, C:N and C:P ratios decreased with increasing latitude and altitude and decreasing MAT and mean annual precipitation at the regional scale (Fang et al., 2019; Zhang et al., 2021a). Salt stress can impose physiological constraints on plants, including nutrient imbalance, osmotic stress, and photosynthetic inhibition, thereby affecting plant growth (Chaves et al., 2009; Rong et al., 2015; Sun et al., 2017). Compared with extensive studies conducted on plant C, N, and P stoichiometry variations with the abovementioned environmental factors, however, relatively few studies have focused on their variations with salinity stress.

For instance, recent studies showed that plant C concentration and C:N ratio decreased as soil salinity increased (Song et al., 2015; Wang et al., 2015), whereas Rong et al. (2015) found no significant relationships between leaf C concentration and C:N ratio and soil salinity. Some studies showed that leaf P concentration decreased, whereas leaf N concentration and N:P ratio increased with salinity stress (Patel et al., 2010; Song et al., 2015). However, other studies found that leaf N and P concentrations both decreased with increase in soil salinity (Ramoliya et al., 2006; Sun et al., 2017). Also, studies have shown that the patterns of N and P concentrations in response to soil salinization are species specific (Loupassaki et al., 2002). Although the effects of salinity stress on plant ecophysiology have been extensively studied, the effects of soil salinity on plant C, N, and P stoichiometries are seldom studied (Sun et al., 2017). Moreover, there was little consensus on the effects of salinity stress on plant C, N, and P stoichiometries, and these studies mostly focused on the leaf level. Therefore, exploring the pattern of the above- and below-ground parts C, N, and P stoichiometry variations with soil salinity can provide new insights into the effects of salinization on the nutrient cycle and the community assembly process.

Plants have different resource utilization strategies at different growth stages (Liu and Wang, 2021). Specifically, nutrients are mainly transported for new tissue development to meet the rapid growth rate during spring seasons, while they are transported to fruits and seeds to produce offspring during autumn and to roots to ensure survival and growth in the following year (Åoren, 1988). Therefore, the C, N, and P stoichiometries of plants would also vary with the growing seasons (Sardans and Peñuelas, 2012; Li et al., 2017b). For instance, leaf N and P concentrations of woody species decreased over the growing seasons, whereas the C:N, C:P, and N:P ratios showed the opposite trend (Dong et al., 2021). Similarly, the leaf N and P concentrations of herbs were significantly higher in the early growth season than in other growing seasons (Wu et al., 2010; Liu et al., 2020; Xiong et al., 2020). In contrast, Huang et al. (2019) showed that riparian plants exhibited lower leaf N and P concentrations and higher leaf C concentration and ratios of C:N, C:P, and N:P in spring than in autumn. However, most studies have focused on the characteristics of leaf C, N, and P stoichiometry during the peak growth period, but ignored the seasonal dynamics of the element stoichiometric characteristics in leaves as well as other parts (especially the belowground part).

Plant C, N, and P stoichiometries are linked to carbon assimilation capacity, nutrient limitation status and performance of plants (Aerts and Chapin, 2000; Hessen et al., 2007; He et al., 2008). Specifically, C:N and C:P ratios can reflect the nutrient utilization efficiency and carbon assimilation rate of plants (Huang et al., 2019). Comparatively, the N:P ratio of plants can reflect dynamic balance between plant nutrition requirement and soil nutrients, and therefore can be regarded as an indicator of soil nutrient limitation (Koerselman and Meuleman, 1996; Herbert et al., 2003; Güsewell, 2004). Generally, common species are finely tuned and have significant growth advantages with high efficiency in exploiting resources in the given environment, while the rare species are likely transients struggling for success for the available conditions (Umaña et al., 2015). Species relative abundance is usually used to distinguish the common and rare species (Mouillot et al., 2013). Therefore, plant C, N, and P stoichiometries should also be related to the relative abundance of the species. To the best of our knowledge, however, no studies have conducted to determine the relationships between species relative abundance and plant C, N, and P stoichiometries. Trait variations within species can enable plants to adapt to varying environmental conditions and alter their interactions with other species, and thus could be crucial for understanding community dynamics (Bolnick et al., 2003; Garzón et al., 2011). Given the assumption that intraspecific variation arising from phenotypic plasticity is much lower than interspecific variation (Garnier et al., 2001), interspecific variation in plant traits has long been considered to be the cornerstone of ecosystem function and community assembly (Adler et al., 2013; Laughlin and Messier, 2015). Recent studies have found that intraspecific variation in traits can be similar to or even greater than the interspecific variation (especially for leaf nutrient concentrations) and is of great significance in predicting the performance of plant communities in response to environmental changes (Fajardo and Siefert, 2016; Pérez-Ramos et al., 2019; Lin et al., 2020). Furthermore, very few studies have examined the links between species relative abundance and the intraspecific variation of plant C, N, and P stoichiometries. Therefore, linking the species relative abundance to plant C, N, and P stoichiometries and their intraspecific variations would help to understand the different strategies between the common and rare species as well as the community assembly process.

The Xiaokai River, located in northeast Shandong Province, China, is a large national Yellow River irrigation area. In this area, riverbeds and tablelands are distributed in strips with scattered shallow saucer-type depressions. The hills and depressions are characterized by gentle slopes, forming a micro-relief terrain alternating with hills, slopes, and depressions. Surface and groundwater runoff in low-lying areas are sluggish and vulnerable to waterlogging and salinity changes. The irrigation area is flat with gentle slope, and the natural terrain is high in the south and low in the north. As a result, salinization of the Xiaokai River irrigation area gradually increases from south to north. This presents a suitable opportunity to explore the variation patterns in the C, N, and P stoichiometric characteristics of plant communities along the soil salinity gradient. Therefore, this study aimed to: (1) determine the variation patterns in both above- and below-ground parts C, N, P stoichiometries of plant community in response to a salinity gradient; (2) identify the dominant soil factors affecting above- and below-ground parts C, N, P stoichiometries of plant community under salinity stress; and (3) examine the relationships between species relative abundance and the above- and below-ground parts C, N, P stoichiometries and their intraspecific variations under salinity stress. Considering that the degree of soil salinization has increased due to rises in sea level and increase of drought incidences (Aragüés et al., 2015; Sun et al., 2017; Cai et al., 2021; Wang et al., 2021), the findings of this study may aid in an improved understanding of the carbon allocation processes in plant communities and better predict their responses to global changes.

## 2 Materials and methods

### 2.1 Site description

The Xiaokai River irrigation area (117°42′–118°04′ E, 37°17′–38°03′ N) adopts river water diversion without a dam on the bank. However, diverting water from the Yellow River inevitably carries sand, which leads to the continuous silting of sediment in the irrigation area. Therefore, the main canal in the irrigation area was designed based on multiple years of statistical data of the Yellow River bottom elevation and water level in front of the sluice, and large gradient and long-distance sediment transport were adopted. The trunk canal of Xiaokai River is 91.5 km long: the sand transport channel is 51.3 km long, the sand settling basin is 4.16 km long, and the water transport channel is 36.04 km long.

The Xiaokai River irrigation landform belongs to the accumulation plain area in the hinterland of the Yellow River Delta, China. Its elevation is generally below 50 m. The irrigated area falls within the temperate monsoon climate zone, which has four distinct seasons with rain and heat at the same time. Rainfall in the irrigation area varies greatly annually and is unevenly distributed. The annual average rainfall is 575.2 mm, and the annual average temperature is 12.3°C. The annual frost-free period is 210 d on average, and the annual light hours are 2400–2700 h. The soil is mostly fluvo-aquic, and the surface soil texture can be roughly divided into four categories: sandy soil, sandy loam, clay loam, and clay.

### 2.2 Experimental design

**Figure 1:**
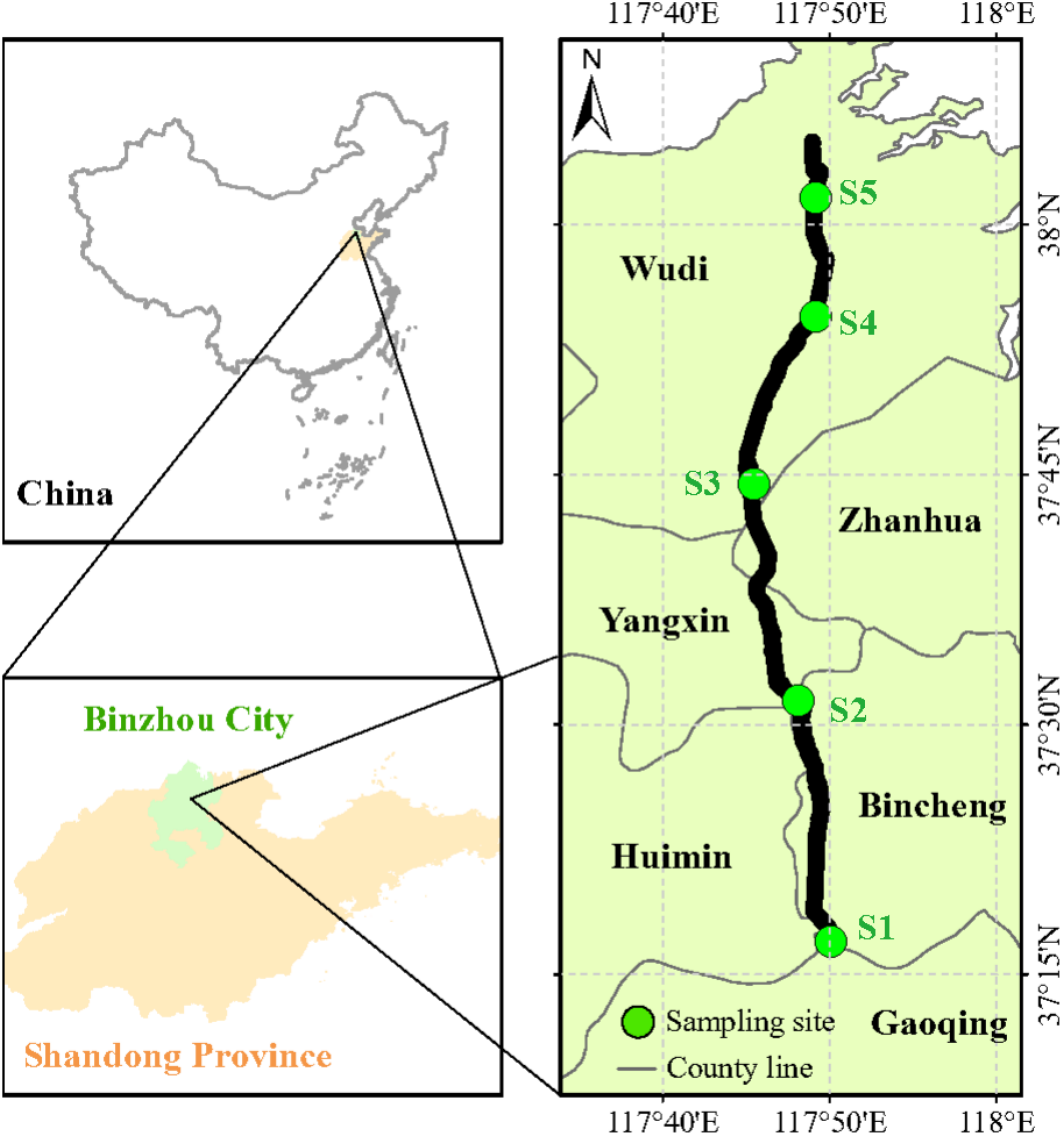
The location of the study area and distribution of sampling sites. S1, very low salinity; S2, low salinity; S3, medium salinity; S4, high salinity; S5, very high salinity.

Plant and soil sampling was carried out in late-May (early growth stage) and mid-September 2021 (late growth stage). Along the Xiaokai River irrigation area, we selected five sampling sites from south to north (one sampling site was set up around, two before and two after the sand settling basin) (**Figure 1**). With the decrease in terrain (from south to north), the degree of salinity increased gradually across the five sampling sites (S1: very low salinity; S2: low salinity; S3: medium salinity; S4: high salinity; S5: very high salinity). See **Figure S1** for the variations of soil pH and electrical conductivity along the Xiaokai River irrigation. Five quadrats (2 × 2 m) were randomly set at each sampling site, and the height, coverage, and number of clusters of all herbs in each quadrat were recorded. Then, the above- and below-ground parts of each species were harvested, and one soil core (0–20 cm) was collected from each quadrat. The plant samples were dried to a constant weight at 65°C. The soil samples were air-dried, and the remaining roots and stones were manually removed. In total, 30 (or 226) and 37 (or 306) species (or above- and belowground part samples) were collected in the early and late growth stages, respectively. For C, N, and P analysis, plant samples were ground in a ball mill (WS-MM301; Retsch, Haan, Germany) and soil samples were ground to a fine powder to pass through a 0.15 mm sieve. Plant and soil C and N concentrations were measured by combustion using an elemental analyzer (vario MACRO cube, Germany). Plant and soil P concentrations were determined using an inductive coupled plasma emission spectrometer (iCAP7600, USA) after HNO_3_ digestion of the plant samples and HNO_3_–HF digestion of the soil samples. Soil pH and electrical conductivity were determined in a 1:2.5 mixture of air-dried soil and distilled water using a glass electrode pH meter (S40, Mettler Toledo, Switzerland) and a conductivity meter (DDS-11A, Leici, China), respectively.

### 2.3 Data analysis

One-way ANOVA was used to analyze the differences in C, N, and P concentrations and their ratios in the plant and soil samples across different sampling sites. An independent sample t-test was used to analyze the differences in above- and below-ground parts C, N, and P stoichiometries of plants in the same sampling season and between sampling seasons in the same plant tissues. Pearson’s correlation was used to test the relationships between and across the above- and below-ground parts C, N, and P stoichiometries of plants. All the above statistical analyses were performed using SPSS (2010, v.19.0; SPSS Inc., Chicago, IL, USA). The RDA-ordination biplot was used to examine the relationships between the plant C, N, and P stoichiometries and the soil properties using the CANOCO software for Windows (ver.5.0, Ithaca, NY, USA).

In each quadrat, the community weight mean (CWM) of plant C, N, and P stoichiometries was calculated in each growth stage (or pooling the two growth stages). First, the weight of each herb species in each quadrat was calculated by dividing their coverage by total plant coverage. Second, the CWM of plant C, N, and P stoichiometries of each quadrat was calculated as the sum of the product of C, N, and P concentrations and their ratios for each species and their weight. The distance of a species from the CWM (ΔCWM) for plant C, N, and P stoichiometries was calculated as the absolute difference between the CWM value and the species-median C, N, and P concentrations and their ratios for each species. A small distance value indicated that the species is close to the average trait value of the community, whereas a higher distance value indicates that the species is in an extreme position in the trait distribution of the community (Umaña et al., 2015). The coefficient of variation (CV) was calculated to characterize the intraspecific variation in plant C, N, and P stoichiometries, which was calculated as CV= 100 × standard deviation of plant C, N, and P stoichiometries divided by the mean values of each species across the sampling sites. The relative species occurrence frequency across the sampling sites was used to represent the species relative abundance (Klanderud and Totland, 2005). Then, the Pearson’s correlation coefficients between species relative abundance and the ΔCWM and the intraspecific variation of plant C, N, and P stoichiometries were calculated using SPSS. When the sampling number of a species in each growing season (or pooling the two growth stages) was more than three, the species would be considered into the CWM calculation. See **Table S1** for details regarding these species.

## 3 Results

### 3.1 Variation patterns in C:N:P stoichiometry of the herbaceous community

**Figure 2:**
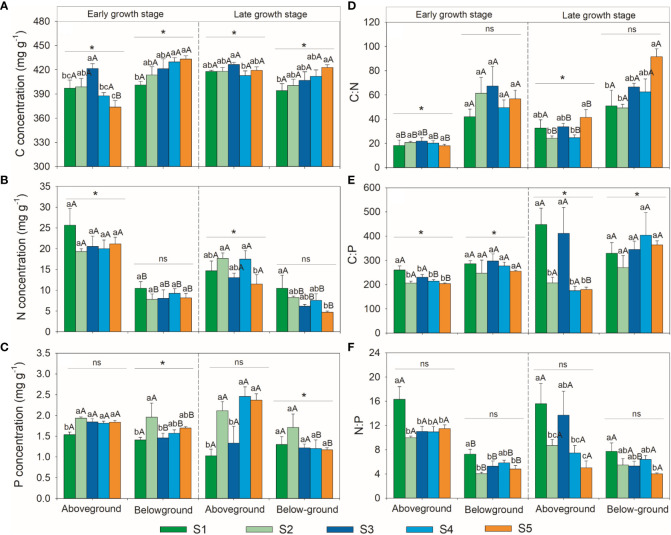
Variations patterns of herbaceous community C, N, P stoichiometries. **(A)** variation pattern of C concentration; **(B)** variation pattern of N concentration; **(C)** variation pattern of P concentration; **(D)** variation pattern of C:N ratio; **(E)**, variation pattern of C:P ratio; **(F)** variation pattern of N:P ratio. S1, very low salinity; S2, low salinity; S3, medium salinity; S4, high salinity; S5, very high salinity. Different uppercase letters indicate significant differences between above- and below-ground parts of the same growth stage. Different lowercase letters indicate significant differences amongst plots in the same part within growth stage. * and ns indicate significant and no significant differences in the same part between growth stages, respectively.

The C, N, P stoichiometries of the above- and below-ground parts showed different variation patterns with soil salinity (**Figure 2**). The aboveground part C concentration first increased and then decreased, whereas that of the belowground part gradually increased with soil salinity in the early growth stage; the aboveground part C concentration increased slightly and then decreased, while that of the belowground part gradually increased with soil salinity in the late growth stage. Both the above- and below-ground parts N concentrations showed no significant changes with soil salinity in the early growth stage; the aboveground part N concentration first remained stable and then decreased, while that in the belowground part gradually decreased with soil salinity in the late growth stage. The aboveground part P concentration first increased and then remained stable, whereas that in the belowground part first increased and then decreased and remained stable with soil salinity in the early growth stage; the aboveground part P concentration first increased and then decreased and increased again, while that of the belowground part showed no significant change with soil salinity during the late growth stage. Both above- and below-ground parts C:N ratios did not change significantly with soil salinity in the early growth stage; the aboveground parts C:N ratio first remained stable and then increased, while that in the belowground part increased gradually with soil salinity in the late growth stage. The aboveground part C:P ratio first decreased and then remained stable, while the belowground part C:P ratio did not change significantly with soil salinity in the early growth stage; the aboveground part C:P ratio first decreased and then increased and decreased again, while the belowground part C:P ratio showed no significant changes with soil salinity in the late growth stage. With the increase in salinity, the above- and below-ground part N:P ratio first decreased and then remained stable in the early growth stage, while both tended to decrease in the late growth stage.

The C, N, P stoichiometries of the above- and below-ground parts also varied with growth seasons (**Figure 2**). The aboveground part C concentration in the early growth stage was significantly lower than that in the late growth stage, whereas that of the belowground part showed the opposite trend. The aboveground part N concentration in the early growth stage was significantly lower than that in the late growth stage, but that of the belowground part showed no significant differences between the growth stages. The aboveground part P concentration showed no significant differences between growth stages, whereas that of the belowground part was significantly higher in the early growth stage than in the late growth stage. The aboveground part C:N ratio in the early growth stage was significantly lower than that in the late growth stage, whereas the belowground part C:N ratio was not significantly different between the growth stages. Both the above- and below-ground parts C:P ratios in the early growth stage were significantly lower than those in the late growth stage, and both the above- and below-ground parts N:P ratios showed no significant differences between the early and late growth stages.

### 3.2 Relationships between above- and below-ground parts C, N, and P stoichiometries of the herbaceous community

**Figure 3:**
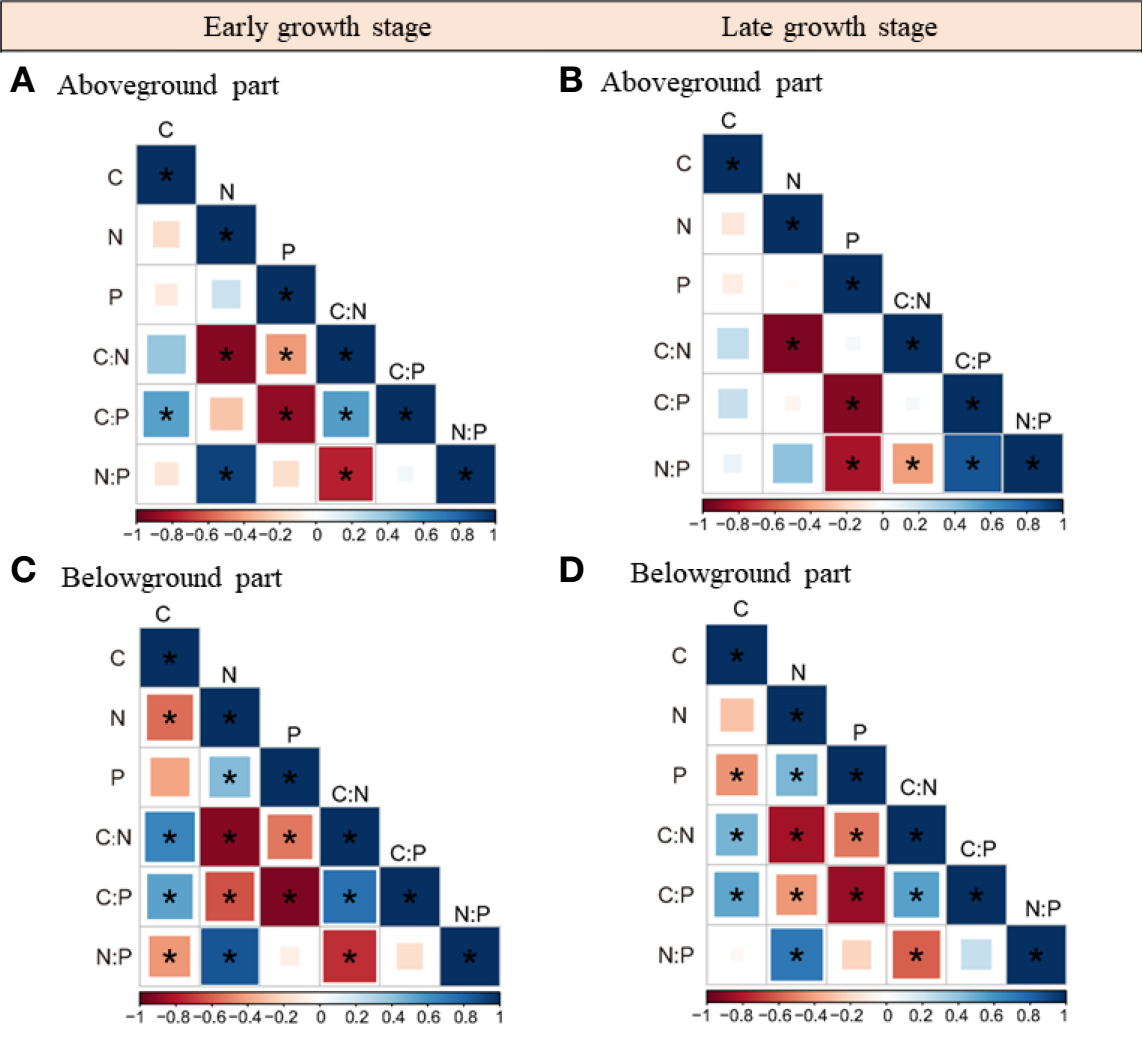
Relationships within the above- and belowground parts C, N, P stoichiometries in the early **(A, B)** and late growth stages **(C, D)**. *, represents significant correlations at the 0.05 level.

The relationships among herbaceous community C, N, and P stoichiometries differed between plant tissues and growth stages (**Figures 3**, **S2**). For instance, the N and P concentrations as well as the C concentration and the C:N ratio were not correlated in the aboveground part, whereas they were closely associated in the belowground part. Also, we found that plant C, N, and P stoichiometries across different plant tissues were closely linked and their relationships varied with sampling seasons (**Tables 1**; **S2**). For example, the aboveground part C concentration was closely related to the C, N, and P stoichiometries of the belowground part in the early growth stage, whereas no correlation was found between them in the late growth stage. The aboveground part P concentration was closely related to the P concentration and C:P ratio of the roots in the early growth stage, whereas it was correlated with the N:P ratio of the belowground part in the late growth stage.

**Table 1:** Relationships between the above- and below-ground parts C, N, P stoichiometries.

The early growth stage							The late growth stage					
Parameter	AC	AN	AP	AC:N	AC:P	AN:P	AC	AN	AP	AC:N	AC:P	AN:P
BC	0.079	-0.289	0.082	0.155	-0.105	-0.316	0.104	-0.035	0.053	0.013	-0.131	-0.147
BN	**-0.572**	**0.657**	0.173	**-0.671**	*-0.389*	**0.587**	0.086	**0.480**	-0.273	**-0.439**	*0.385*	**0.622**
BP	**-0.502**	0.065	**0.495**	-0.254	**-0.608**	-0.106	-0.079	*0.371*	0.117	-0.294	-0.130	0.073
BC:N	**0.628**	**-0.497**	-0.073	**0.540**	0.314	**-0.468**	-0.128	**-0.515**	0.147	**0.502**	-0.233	**-0.495**
BC:P	**0.602**	-0.240	**-0.421**	*0.393*	**0.581**	-0.087	-0.054	-0.165	-0.160	0.124	0.121	-0.007
BN:P	*-0.340*	**0.693**	-0.170	**-0.583**	-0.016	**0.747**	0.106	*0.374*	**-0.409**	*-0.389*	**0.510**	**0.682**

Fond in bold when *P* < 0.05 and in italic when *P* < 0.1. AC, aboveground part C concentration; AN, aboveground part N concentration; AP, aboveground part P concentration; AC:N, aboveground part C:N ratio; AC:P, aboveground part C:P ratio; AN:P, aboveground part N:P ratio; BC, belowground part C concentration; BN, belowground part N concentration; BP, belowground part P concentration; BC:N, belowground part C:N ratio; BC:P, belowground part C:P ratio; BN:P, belowground part N:P ratio.

### 3.3 Relationships between herbaceous community C:N:P stoichiometry and soil properties

**Figure 4:**
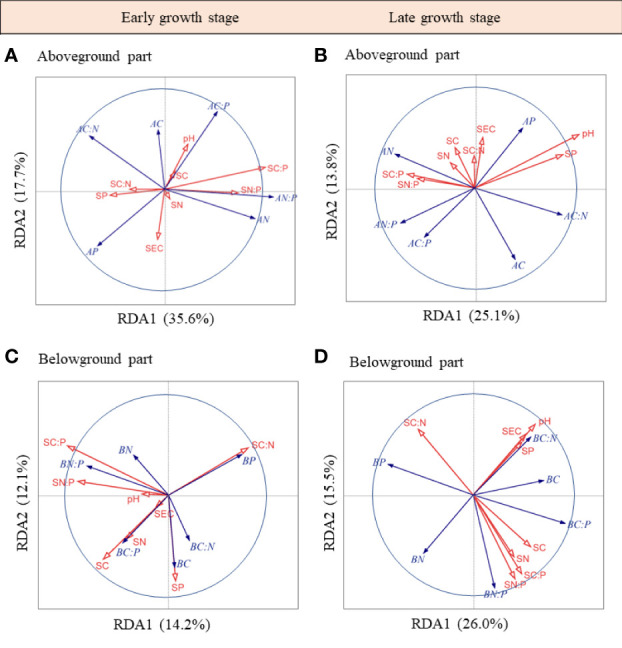
RDA-ordination biplot of the above- and belowground parts C, N, P stoichiometries and soil properties in the early **(A, B)** and late growth stages **(C, D)**. AC, aboveground part C concentration; AN, aboveground part N concentration; AP, aboveground part P concentration; AC:N, aboveground part C:N ratio; AC:P, aboveground part C:P ratio; AN:P, aboveground part N:P ratio; BC, belowground part C concentration; BN, belowground part N concentration; BP, belowground part P concentration; BC:N, belowground part C:N ratio; BC:P, belowground part C:P ratio; BN:P, belowground part N:P ratio; SC, soil C concentration; SN, soil N concentration; SP, soil P concentration; SC:N, soil C:N ratio; SC:P, soil C:P ratio; SN:P, soil N:P ratio; pH, soil pH; SEC, soil electrical conductivity.

The RDA ordination biplot showed that the dominant determining soil factor of herbaceous community C, N, and P stoichiometries varied with plant tissues and sampling seasons (**Figure 4**). During the early growth stage, soil C:P ratio, N and P concentrations, and soil electrical conductivity were the major factors of the aboveground part C, N, and P stoichiometries (**Figure 4A**), which contributed 39.0%, 17.2% and 14.0%, and 12.7% variations in these factors, respectively; soil C:P ratio was the major factor of the belowground part C, N, and P stoichiometries (**Figure 4B**), which contributed 32.1% variations in these factors. During the late growth stage, soil pH and C:N ratio were the major factors of the aboveground part C, N, and P stoichiometries (**Figure 4C**), which contributed 41.2% and 18.7% variations in these factors, respectively; soil P, pH, and C:N ratio were the major factors in the belowground part C, N, and P stoichiometries (**Figure 4D**), which contributed 28.6%, 22.0%, and 21.9% of the variations in these factors, respectively. Upon pooling the early and late growth stages, the soil pH, C:P ratio, soil electrical conductivity, and C:N ratio were found to be the major factors affecting the aboveground part C, N, and P stoichiometries (**Figure S3A**), which contributed 36.1%, 20.2%, 13.5%, and 12.1% of the variations in these factors, respectively; soil C:P ratio, pH, and P concentration were the major factors of the belowground part C, N, and P stoichiometries (**Figure S3B**), which contributed to 31.3%, 20.9%, and 16.0% of the variations in these factors, respectively.

### 3.4 Relationships between species relative abundance and the C:N:P stoichiometry of herbs and its intraspecific variations

**Figure 5:**
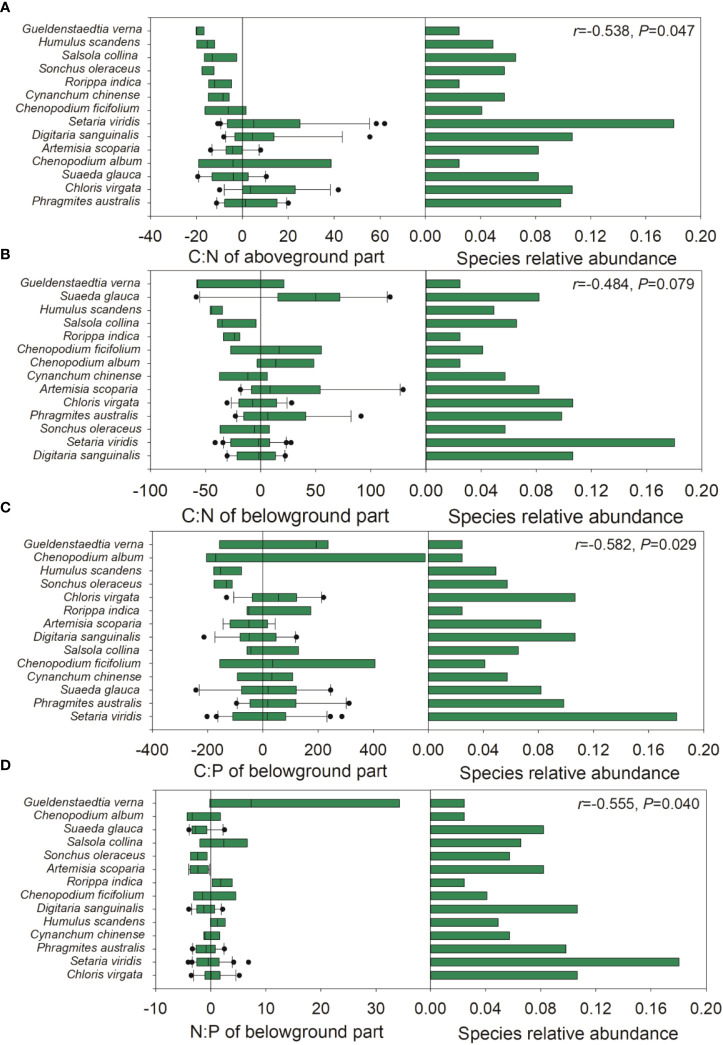
The relative position of species C:N:P stoichiometries distribution across sampling sites in the late growth stage. **(A)**, relative position of the C:N of aboveground part; **(B)**, relative position of C:N of belowground part; **(C)** relative position of N:P ratio of belowground part; **(D)** relative position of N:P of belowground part across sampling sites. Left panel: the x-axis represents the difference between the median C, N, P stoichiometries for each species and the community-weighted mean (CWM) C, N, P stoichiometries for the entire plant community. The y-axis arrays species from bottom to top based on their distance how close to the CWM value. Each boxplot represents the distribution of C, N, P stoichiometries of each species. Right panel: species relative abundance across sampling sites. The *r*-value of Pearson correlation analysis of the absolute values of the differences between the median C, N, P stoichiometries of each species and the CWM value of the entire community against species relative abundance is provided at the upper right.

**Figure 6:**
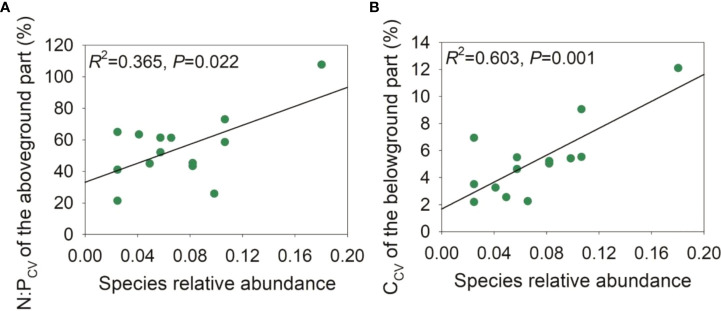
Relationships between species relative abundance and intraspecific variation of plant C:N:P stoichiometries in the late growth stage. **(A)**, relationship between intraspecific variation of N:P (N:P_CV_) of the aboveground part in relation to species relative abundance; **(B)**, relationship between intraspecific variation of C concentration (C_CV_) of the belowground part in relation to species relative abundance.

During the late growth season, the species relative abundance was negatively correlated with the ΔCWM of the C:N ratio of the aboveground part and the C:P and N:P ratios of the belowground part, and moderately negatively correlated with the ΔCWM of the C:N ratio of the belowground part (**Figure 5**). Upon pooling the early and late growth stages, the species relative abundance was negatively associated with the ΔCWM of P and the C:P ratio of the aboveground part, and moderately negatively associated with the ΔCWM of P concentration (**Figure S4**). During the late growth season, we found a positive correlation between the species relative abundance and the intraspecific variation in the N:P ratio of the aboveground part and the C concentration of the belowground part (**Figure 6**). Upon pooling the early and late growth stages, there was a positive correlation between the species relative abundance and the C concentration of the belowground part (**Figure S5**). Given that soil pH did not show significant changes with soil salinity and the relative fewer species compared with the late growth stage (**Figure S1**, **Table S1**), no associations were found between the species’ relative abundance and the ΔCWM and the intraspecific variations in plant C, N, and P stoichiometries during the early growth stage.

## 4 Discussion

### 4.1 Variation patterns of C:N:P stoichiometry in the herbaceous community

Consistent with previous studies, the plant C concentration of aboveground parts first remained unchanged or slightly increased, and then gradually decreased with soil salinity. This may be because soil salinity stress could restrain plants photosynthetic abilities by decreasing stomatal conductance and leaf water potential (Wang et al., 2015; He et al., 2016; Cao et al., 2020). Meanwhile, our results showed that plant C concentrations in belowground parts gradually increased with increasing salinity, indicating that plants would allocate more carbon to the belowground part to adapt to harsh environments. This implied that increasing the C concentration and some C-rich compounds could help plants resist and adapt to harsh environments (Sardans and Peñuelas, 2014; Li et al., 2021). To avoid severe salinity stress in shallow soil, plants can enhance root growth and extension to absorb water and nutrients from deeper soil (Wang et al., 2015). Therefore, starch in the aboveground part is transferred to the belowground part for storage, which is conducive to sprouting new roots and improving root activity (Yang et al., 2018). Consistent with previous studies (Zhang et al., 2021c; Xiong et al., 2022), the coefficient of variation in plant C concentration was lower than that in N and P concentrations (**Table S3**), indicating that plant C concentration is relatively stable under salt stress. This may be due to the different C assimilation and N and P uptake pathways: plant C mainly comes from CO_2_ assimilation from the atmosphere during photosynthesis, whereas plant N and P are mainly derived from soil (Berman-Frank and Dubinsky, 1999). Soil N and P concentrations both showed an increasing trend with soil salinity (although some cases are not significant, data not shown), however, plant N and P concentrations showed different trends: plant N concentration remained unchanged or decreased, while P concentration remained unchanged or increased with increasing salinity. This may be explained by the fact that soil P concentration strongly influences plant tissue P concentration, while soil N concentration has little effect on plant tissue N concentration (Li et al., 2019; Liu et al., 2019).

According to the growth rate hypothesis, organisms allocate large amounts of P to ribosomal RNA during periods of rapid growth to facilitate quick synthesis of large amounts of protein by ribosomes (Aerts, 1996). Therefore, higher plant growth rates are generally associated with lower C:N and C:P ratios (Elser et al., 2000; Vrede et al., 2004; Hessen et al., 2007). In this study, the C:N ratio increased with soil salinity, indicating that salinization could inhibit plant growth. However, the C:P ratio of the aboveground part decreased with soil salinity, which suggested a higher growth rate of plants grown in more saline areas. This indicates that the growth rate hypothesis may not be generally applicable to regions with high environmental heterogeneity. The N:P ratio of plants is regarded as an indicator of soil nutrient limitation (Herbert et al., 2003; Drenovsky and Richards, 2004). Generally, N:P ratio < 14 indicates that plant growth is mainly limited by N, N:P ratio > 16 indicates that plant growth is mainly limited by P, and 14 < N:P ratio < 16 indicates N and P colimitation (Koerselman and Meuleman, 1996; Aerts and Chapin, 2000; Güsewell, 2004). In this study, N:P ratios were all less than 14 except for sampling site S1, and tissue N:P ratios gradually decreased with increasing salinization, indicating that N limitation was gradually aggravated along the Xiaokai River irrigation area. However, aboveground part P concentration increased with increasing soil P concentration especially in the late growth stage (**Figure 4**), suggesting that plants were probably limited by soil P (Han et al., 2005). This may be explained by the fact that plants can store higher levels of inorganic P in the leaf cytoplasm and vacuoles by luxury consumption with increasing soil P concentration (Close and Beadle, 2004; Mayor et al., 2014; Yan et al., 2015). Therefore, our study area might be constrained by both N and P. The decline in N:P ratios does not necessarily indicate the absence of P limitation (Mayor et al., 2014), and caution should be exercised when using the N:P ratio to characterize plant nutrient restriction. We also found that the plant N:P ratio was more closely related to N than to P, which also suggested that plant growth was more limited by N than by P (Wang et al., 2015). Therefore, the optimal application of N fertilizer may be practical to promote plant growth in downstream areas, and N-fixing plants of the legume family could also be introduced to improve soil fertility. Moreover, the increase of N and P fertilizer can alleviate the damage of salt stress on plants, and improve the N and P nutrient status in plants. This may also be the reason that soil N and P concentration showed increase trend with soil salinity.

The aboveground part had higher N, P, and N:P ratio but lower C:N and C:P ratios than those of the belowground part, especially in more heavily salinized regions. This suggests that different plant tissues distribute the available nutrients in different ways under environmental changes (He et al., 2015). Under drought or soil salinity stress, plants can regulate the limited nutrients between plant tissues, especially with a larger proportion of N and P allocated to the photosynthetic tissues than to the non-photosynthetic tissues, to achieve optimal energy production and maintain plant growth (Reich and Oleksyn, 2004; Zhang et al., 2021c). In addition, the demand for N in rubisco results in a higher N concentration per unit C loss for leaves than for roots during dark respiration and could also lead to leaves requiring more P than roots, and therefore results in differences in N to P stoichiometry between leaves and roots (Reich et al., 2008; Yan et al., 2016; Liu et al., 2019). These findings highlight the importance of determining C, N, and P stoichiometries in both photosynthetic and non-photosynthetic tissues of plants in response to environmental stress.

Consistent with previous studies (Dong et al., 2021; Li et al., 2022), we found that seasonal variation of the structural substance C concentration was lower than that of N and P concentrations. Nutrient elements (i.e. N and P) are mainly transported to photosynthetic tissues to meet the demand for rapid growth during the early growth stage. In this period, photosynthetic tissues have the ability to divide rapidly, and realize rapid cell proliferation by absorbing a large amount of N and P substances to synthesize proteins and nucleic acids (Wu et al., 2010). As a result, the aboveground part N and P concentrations were high, while the C:N and C:P ratios were low. During the late growth stage, high temperatures increase soil evaporation and plant transpiration. To enhance drought and salt tolerance, plants tend to increase their aboveground part C concentration, which resulted in an increase in their C:N and C:P ratios. Accordingly, the plant growth rate slowed down and water consumption decreased during the late growth stage. Moreover, N and P concentrations could be diluted in plant individuals due to the rapid increase in plant size and biomass (Sardans and Peñuelas, 2008). Also, the decrease of N and P concentrations in the late growth stage may be caused by the redistribution of nutrients to reproductive tissues (e.g. flower) (Liu et al., 2015). Correspondingly, the aboveground part C:N and C:P ratios increased during the late growth stage, which indicated a higher nutrient use efficiency (Huang et al., 2019). Li et al. (2021) found that there was a negative correlation between C:N ratio and the characteristic parameters δ^13^C of water use efficiency. Similarly, Salazar-Tortosa et al. (2018) showed that plants cannot simultaneously optimize water and nitrogen use efficiency in natural ecosystems, and their utilization strategy is to effectively utilize one resource at the expense of another. Collectively, these results indicated that variations in plant C, N, P stoichiometries between different growth stages might be related to the trade-offs between water and nutrient use efficiency.

### 4.2 Relationships between herbaceous community C:N:P stoichiometries and soil properties

Soil pH, reflecting salinity and alkalinity, is an important determinant of microbial community composition and activity in plant communities and thus affects many ecological processes (e.g. soil respiration, microbial C use efficiency, and N and P mineralization) (Bååth and Anderson, 2003; Kuzyakov and Blagodatskaya, 2015; Zhou et al., 2017). Generally, plant carbon assimilation decreases under salinity stress (He et al., 2016; Cao et al., 2020; Zhang et al., 2021c). However, we found that the belowground part C concentration was positively related to soil pH (**Figures 2**, **4**). This might be because plants increase the fractions of decay-resistant compounds (e.g. lignin, cellulose, and tannin) to increase their resistance (Sardans and Peñuelas, 2014; He et al., 2015). In our study, the soil pH was between 7.5 and 8.5 (**Figure S1**). Therefore, the salinity stress of our study region was medium based on the standards for the second national soil census (National Soil Census Office of China, 1992). Then, the positive relationship between belowground part C concentration and soil pH may also be explained by the “growth stimulation” at optimum salt concentration since plants would enhance solute uptake required to induce cell expansion to maintain the osmotic potential in their tissues (Parida et al., 2004; Sun et al., 2017).

Higher C:N and C:P ratios can reflect higher carbon assimilation rate and nutrient utilization efficiency of plants (Huang et al., 2019). We found that the organ C:N ratio increased with soil pH, indicating that the N utilization efficiency increased with salinity. This may be due to the fact that salinity stress could affect plants photosynthetic related traits and disrupt their ion balance and protein synthesis, and thus decreasing plant C and N concentrations (Qiang et al., 2018). Therefore, plants can enhance their adaptability to salinity stress by increasing their N utilization efficiency (Zhou et al., 2021). However, P utilization efficiency did not always increase with soil salinity in our study. This may be explained by the fact that the study area is more limited by N than P, and therefore plants improved the efficiency of the more restricted elements but retained a certain amount of P to adapt to the environment. Similarly, previous studies have shown that plants can store higher levels of inorganic P in the leaf cytoplasm and vacuoles by luxury consumption when the soil P concentration is high (Mayor et al., 2014; Yan et al., 2015). By increasing tissue P concentration, plants can promote carbohydrate metabolism and increase the concentration of soluble sugar and phosphate (He et al., 2022). This can make their intracellular protoplasm buffer against changes in acidity and alkalinity, thus improving their adaptability to salinity stress (Kornberg et al., 1999; Werner et al., 2007).

In terrestrial ecosystems, plant P concentration is primarily determined by soil parent material and the degree of rock weathering (Vitousek et al., 2010; Fan et al., 2015; Yang et al., 2016). Therefore, organ P concentration is positively related to soil P concentration, which has been revealed by a large number of previous studies (Han et al., 2005; Yang et al., 2016; Liu et al., 2019). However, soil N had little effect on plant N concentration, which has also been confirmed in earlier studies (He et al., 2015; Liu et al., 2019; Shi et al., 2021). This may be explained by the fact that plant N concentration is mainly affected by plant functional groups, however, plant P concentration and N:P ratio are mainly determined by climatic factors and soil P concentration (Liu et al., 2019). These results reveal that soil P might reflect plant nutrient status better than soil C and N in salinized ecosystems, and thus indicating that plant P could play a greater role than plant N in plant growth and ecosystem development (Reich et al., 2009; Vitousek et al., 2010; He et al., 2015).

Soil C:P ratio has an important effect on plant growth and development and is a useful indicator to determine the source of organic matter (Tessier and Raynal, 2003; Bui and Henderson, 2013). A high soil C:P ratio restricts the decomposition of organic matter and is not conducive to plant growth (Ren et al., 2007). In this study, we found that the soil C:P ratio was the main factor affecting plant element stoichiometry for the aboveground part, belowground part, and whole plant level in the early growth stage. The negative relationship between the soil C:P ratio and plant P can be explained by the fact that a higher soil C:P ratio would cause microorganisms to be limited by soil P during the decomposition of organic matter and compete with plants for soil P, which is not conducive to nutrient absorption by plants (Zeng et al., 2015). Soil C:N ratio can reflect the mineralization and humification of soil organic matter, and soil organic matter with a low C:N ratio usually leads to faster decomposition by microorganisms, which can provide nitrogen input back into the ecosystem (Cornwell et al., 2008). Therefore, we also found that the soil C:N ratio was the main influencing factor of plant element stoichiometry for the aboveground part in the late growth stage. Our results revealed that the soil C:N and C:P ratios had greater effects than the soil C and N on determining plant nutrients. This may be explained by that plant nutrient concentrations were more sensitive to the supply of soil nutrient ratios rather than the supply of a single nutrient (Bowman and Hurry, 1993).

### 4.3 Relationships between species relative abundance and the C:N:P stoichiometry of herbs and its intraspecific variation

A negative correlation was found between the ΔCWM of plant C, N, and P stoichiometries and species relative abundance in the late growth stage (or pooling the two growth stages), suggesting that the C, N, P stoichiometries of the common species was medium (neither very high nor very low) compared with the rare species. This can be explained by the fact that common species often occupy core positions within the community trait space, whereas rare species are usually peripheral in function (Umaña et al., 2015). A core position indicates that the specific trait value expressed by a species is similar to the average trait value of the entire community. This allows for high efficiency in resource exploitation in a given environment and can lead to significant growth advantages (Muscarella and Uriarte, 2016). Therefore, the positive relationship between plant P and soil salinity might be due to the effect of species turnover from the shared common species (e.g. *Setaria viridi*, *Chloris virgata*, *Metaplexis japonica*) with medium P concentration to salt-tolerant species (e.g. *Phragmites australis*, *Suaeda glauca*) with high P concentration with increasing soil salinity (Luo et al., 2018; Gong et al., 2020). The shift in the plant community toward species with higher nutrient concentrations could increase their competitive advantages under environmental stress (Weih et al., 2011; Sardans and Peñuelas, 2012; Li et al., 2017a; Luo et al., 2018), and the higher nutrient concentrations could help them opportunistically maximize photosynthesis during periods suitable for growth (Farquhar et al., 2002; Weih et al., 2011).

Both species turnover and intraspecific variation could play critical roles in driving community nutrient responses to environmental changes (Violle et al., 2012; Luo et al., 2018; Gong et al., 2020). In this study, the intraspecific variability of the aboveground part N:P ratio and belowground part C showed a positive correlation with species relative abundance in the late growth stage (or pooling the two growth stages). Previous studies have demonstrated that the leaf N:P ratio can be used as an indicator of vegetation composition, function, and nutrient limitation at the community level (Koerselman and Meuleman, 1996; Güsewell, 2004). Thus, the increase in belowground part C concentration and some C-rich compounds could help the plants resist and adapt to harsh environments and benefit them by extending their roots to deeper soils with low salinity (Sardans and Peñuelas, 2014; Yang et al., 2018; Li et al., 2021). However, Umaña et al. (2015) and Jiang et al. (2020) found that common species had a low intraspecific trait variation, indicating a convergent strategy that emphasizes a core physiological connection to the habitat, thus enabling efficient exploitation of available resources (Grime, 2006). The contradictory results might be explained by that our research was conducted in an environment with large environmental heterogeneity, while the above studies were conducted in a smaller homogeneous condition. Consistent with this study, Li et al. (2017c) found that common species tend to have higher intraspecific variation in root traits at the regional scale. This indicates that higher intraspecific trait variation might facilitate greater fitness and survival opportunities in environments with high heterogeneity (Forsman, 2014; González-Suárez et al., 2015). This also supported the research by Umaña et al. (2015), who hypothesized that the association between species relative abundance and intraspecific variation of traits was closely related to the degree of environmental heterogeneity and thus transforming from a negative relationship locally to a positive relationship regionally. As a result, intraspecific variation can promote stability in plant communities by leading to stress adjustment without intense species turnover (Lloret et al., 2012; Jung et al., 2014).

## 5 Conclusion

Our results revealed that plant community C, N, and P stoichiometries and their determining soil properties varied with plant tissues as well as sampling seasons. In this study, the C concentration of belowground part increased with soil salinity. Plant N concentration and C:N ratio tended to decrease with soil salinity, whereas the P concentration, C:P, and N:P ratios showed the opposite trend. The soil C:P ratio and P concentration were the major factors of the C, N, and P stoichiometries in the early growth stage, whereas the soil pH and P concentration were the major factors of the C, N, and P stoichiometries in the late growth stage. Compared with rare species, the C:N:P stoichiometry of common species was medium (neither very high nor very low) which was similar to the average trait value of the entire community. Moreover, the intraspecific variation in the aboveground part N:P ratio and the belowground part C concentration showed a positive correlation with species relative abundance, which indicating that higher intraspecific trait variation might facilitate greater fitness and survival opportunities in environments with high heterogeneity. Our results highlight the importance of intraspecific variation in determining the functional response of plant communities to environmental stress. Whether our results are applicable in a larger scale needs further study. To better understand the adaptive strategies of plant communities to changing environments, further studies should also consider the effect of the climatic factors and determine the C, N, P stoichiometries variations of litter and rhizosphere.

## Data availability statement

The raw data supporting the conclusions of this article will be made available by the authors, without undue reservation.

## Author contributions

PJ designed and implemented the research with input from SF and XZ. PJ conducted field work and performed the experiments. PJ analyzed the data and wrote the paper. All authors contributed to the article and approved the submitted version.

## References

[B1] Adler P. B. Fajardo A. Kleinhesselink A. R. Kraft N. J. (2013). Trait-based tests of coexistence mechanisms. Ecol. Lett. 16, 1294–1306. doi: 10.1111/ele.12157 23910482

[B2] Aerts R. (1996). Nutrient resorption from senescing leaves of perennials: Are there general pat terns. J. Ecol. 84, 597–608. doi: 10.2307/2261481

[B3] Aerts R. Chapin F. S. (2000). The mineral nutrition of wild plants revisited: A re-evaluation of processes and patterns. Adv. Ecol. Res. 30, 1–67. doi: 10.1016/S0065-2504(08)60016-1

[B4] Åoren G. (1988). Ideal nutrient productivities and nutrient proportions in plant growth. Plant Cell Environ. 11, 613–620. doi: 10.1111/j.1365-3040.1988.tb01803.x

[B5] Aragüés R. Medina E. Zribi W. Clavería I. Álvaro-Fuentes J. Faci J. (2015). Soil salinization as a threat to the sustainability of deficit irrigation under present and expected climate change scenarios. Irrigation Sci. 33, 67–79. doi: 10.1007/s00271-014-0449-x

[B6] Bååth E. Anderson T.-H. (2003). Comparison of soil fungal/bacterial ratios in a pH gradient using physiological and PLFA-based techniques. Soil Biol. Biochem. 35, 955–963. doi: 10.1016/S0038-0717(03)00154-8

[B7] Berman-Frank I. Dubinsky Z. (1999). Balanced growth in aquatic plants: Myth or reality? Bioscience 49, 29–37. doi: 10.2307/1313491

[B8] Bolnick D. I. Svanback R. Fordyce J. A. Yang L. H. Davis J. M. Hulsey C. D.. (2003). The ecology of individuals: Incidence and implications of individual specialization. Am. Nat. 161, 1–28. doi: 10.2307/3078879 12650459

[B9] Bowman E. H. Hurry D. (1993). Strategy through the option lens: An integrated view of resource investments and the incremental-choice process. Acad. Manage. Rev. 18, 760–782. doi: 10.5465/amr.1993.9402210157

[B10] Bui E. N. Henderson B. L. (2013). C:N:P stoichiometry in Australian soils with respect to vegetation and environmental factors. Plant Soil 373, 553–568. doi: 10.1007/s11104-013-1823-9

[B11] Cai R. S. Liu K. X. Tan H. J. (2021). Climate change and china’s coastal zones and seas: Impacts, risks, and adaptation. Chin. J. Popul. Resour. Environ. 19, 304–310. doi: 10.1016/j.cjpre.2022.01.003

[B12] Cao J. Wang X. Adamowski J. F. Biswas A. Liu C. Chang Z.. (2020). Response of leaf stoichiometry of *Oxytropis ochrocephala* to elevation and slope aspect. Catena 194, 104772. doi: 10.1016/j.catena.2020.104772

[B13] Chaves M. Flexas J. Pinheiro C. (2009). Photosynthesis under drought and salt stress: regulation mechanisms from whole plant to cell. Ann. Bot. 103, 551–560. doi: 10.1093/aob/mcn125 18662937 PMC2707345

[B14] Close D. C. Beadle C. L. (2004). Total, and chemical fractions, of nitrogen and phosphorus in eucalyptus seedling leaves: Effects of species, nursery fertiliser management and transplanting. Plant Soil 259, 85–95. doi: 10.1023/B:PLSO.0000020942.97995.f3

[B15] Cornwell W. K. Cornelissen J. H. C. Amatangelo K. Dorrepaal E. Eviner V. T. Godoy O.. (2008). Plant species traits are the predominant control on litter decomposition rates within biomes worldwide. Ecol. Lett. 11, 1065–1071. doi: 10.1111/j.1461-0248.2008.01219.x 18627410

[B16] Dong C. Qiao Y. Cao Y. Chen Y. Wu X. Xue W. (2021). Seasonal variations in carbon, nitrogen, and phosphorus stoichiometry of a *Robinia pseudoacacia* plantation on the loess hilly region, China. Forests 12, 214. doi: 10.3390/f12020214

[B17] Drenovsky R. E. Richards J. H. (2004). Critical n: P values: Predicting nutrient deficiencies in desert shrublands. Plant Soil 259, 59–69. doi: 10.1023/B:PLSO.0000020945.09809.3d

[B18] Elser J. J. Fagan W. F. Denno R. F. Dobberfuhl D. R. Folarin A. Huberty A.. (2000). Nutritional constraints in terrestrial and freshwater food webs. Nature 408, 578–580. doi: 10.1038/35046058 11117743

[B19] Elser J. J. Fagan W. F. Kerkhoff A. J. Swenson N. G. Enquist B. J. (2010). Biological stoichiometry of plant production: Metabolism, scaling and ecological response to global change. New Phytol. 186, 593–608. doi: 10.2307/27797587 20298486

[B20] Fajardo A. Siefert A. (2016). Phenological variation of leaf functional traits within species. Oecologia 180, 951–959. doi: 10.1007/s00442-016-3545-1 26796408

[B21] Fan H. Wu J. Liu W. Yuan Y. Hu L. Cai Q. (2015). Linkages of plant and soil C:N:P stoichiometry and their relationships to forest growth in subtropical plantations. Plant Soil 392, 127–138. doi: 10.1007/s11104-015-2444-2

[B22] Fang Z. Li D. Jiao F. Yao J. Du H. (2019). The latitudinal patterns of leaf and soil C:N:P stoichiometry in the loss plateau of China. Front. Plant Sci. 10. doi: 10.3389/fpls.2019.00085 PMC6436477 30949183

[B23] Farquhar G. D. Buckley T. N. Miller J. M. (2002). Optimal stomatal control in relation to leaf area and nitrogen content. Silva Fenn. 36, 625–637. doi: 10.14214/sf.530

[B24] Forsman A. (2014). Effects of genotypic and phenotypic variation on establishment are important for conservation, invasion, and infection biology. P. Natl. Acad. Sci. U.S.A. 111, 302–307. doi: 10.1073/pnas.1317745111 PMC3890895 24367109

[B25] Gargallo-Garriga A. Sardans J. Perez-Trujillo M. Oravec M. Urban O. Jentsch A.. (2015). Warming differentially influences the effects of drought on stoichiometry and metabolomics in shoots and roots. New Phytol. 207, 591–603. doi: 10.1111/nph.13377 25772030

[B26] Garnier E. Laurent G. Bellmann A. Debain S. Berthelier P. Ducout B.. (2001). Consistency of species ranking based on functional leaf traits. New Phytol. 152, 69–83. doi: 10.1046/j.0028-646x.2001.00239.x 35974476

[B27] Garzón M. B. Alia R. Robson T. M. Zavala M. A. (2011). Intra-specific variability and plasticity influence potential tree species distributions under climate change. Global Ecol. Biogeogr. 20, 766–778. doi: 10.1111/j.1466-8238.2010.00646.x

[B28] Gong Y. Ling H. Chen Y. Cao J. Guo Z. Lv G. (2020). N:P stoichiometric changes *via* species turnover in arid versus saline desert environments. Ecol. Evol. 10, 6635–6645. doi: 10.1002/ece3.6395 PMC7381577 32724538

[B29] González-Suárez M. Bacher S. Jeschke J. M. (2015). Intraspecific trait variation is correlated with establishment success of alien mammals. Am. Nat. 185, 737–746. doi: 10.1086/681105 25996859

[B30] Grime J. P. (2006). Trait convergence and trait divergence in herbaceous plant communities: Mechanisms and consequences. J. Veg. Sci. 17, 255–260. doi: 10.1111/j.1654-1103.2006.tb02444.x

[B31] Güsewell S. (2004). N:P ratios in terrestrial plants: Variation and functional significance. New Phytol. 164, 243–266. doi: 10.1111/j.1469-8137.2004.01192.x 33873556

[B32] Han W. X. Fang J. Y. Guo D. L. Zhang Y. (2005). Leaf nitrogen and phosphorus stoichiometry across 753 terrestrial plant species in China. New Phytol. 168, 377–385. doi: 10.1111/j.1469-8137.2005.01530.x 16219077

[B33] He J.-S. Wang L. Flynn D. F. Wang X. Ma W. Fang J. (2008). Leaf nitrogen: phosphorus stoichiometry across Chinese grassland biomes. Oecologia 155, 301–310. doi: 10.1007/s00442-007-0912-y 18278518

[B34] He M. Zhang K. Tan H. Hu R. Su J. Wang J.. (2015). Nutrient levels within leaves, stems, and roots of the xeric species *Reaumuria soongorica* in relation to geographical, climatic, and soil conditions. Ecol. Evol. 5, 1494–1503. doi: 10.1002/ece3.1441 25897388 PMC4395178

[B35] He M. Song X. Tian F. Zhang K. Zhang Z. Chen N.. (2016). Divergent variations in concentrations of chemical elements among shrub organs in a temperate desert. Sci. Rep. 6, 20124. doi: 10.1038/srep20124 26818575 PMC4730183

[B36] He H. Wu N. Liu J. Xu X. (2022). Effects of phosphorus application on the growth and salt resistance of switchgrass under saline alkali conditions. Acta Pratacul. Sin. 31, 64–74. doi: 10.11686/cyxb2021378

[B37] Herbert D. A. Williams M. Rastetter E. B. (2003). A model analysis of n and p limitation on carbon accumulation in Amazonian secondary forest after alternate land-use abandonment. Biogeochemistry 65, 121–150. doi: 10.2307/1469731

[B38] Hessen D. Jensen T. Kyle M. Elser J. (2007). RNA Responses to n- and p-limitation; reciprocal regulation of stoichiometry and growth rate in brachionus. Funct. Ecol. 21, 956–962. doi: 10.2307/4540104

[B39] Huang D. Wang D. Ren Y. (2019). Using leaf nutrient stoichiometry as an indicator of flood tolerance and eutrophication in the riparian zone of the lijang river. Ecol. Indic. 98, 821–829. doi: 10.1016/j.ecolind.2018.11.064

[B40] Jiang P. Wang H. Meinzer F. C. Kou L. Dai X. Fu X. (2020). Linking reliance on deep soil water to resource economy strategies and abundance among coexisting understorey shrub species in subtropical pine plantations. New Phytol. 225, 222–233. doi: 10.1111/nph.16027 31247133

[B41] Jung V. Albert C. H. Violle C. Kunstler G. Loucougaray G. Spiegelberger T. (2014). Intraspecific trait variability mediates the response of subalpine grassland communities to extreme drought events. J. Ecol. 102, 45–53. doi: 10.1111/1365-2745.12177

[B42] Klanderud K. Totland Ø. (2005). Simulated climate change altered dominance hierarchies and diversity of an alpine biodiversity hotspot. Ecology 86, 2047–2054. doi: 10.1890/04-1563

[B43] Koerselman W. Meuleman A. F. M. (1996). The vegetation N:P ratio: a new tool to detect the nature of nutrient limitation. J. Appl. Ecol. 33, 1441–1450. doi: 10.2307/2404783

[B44] Kornberg A. Rao N. N. Ault-Riché D. (1999). Inorganic polyphosphate: A molecule of many functions. Annu. Rev. Biochem. 68, 89–125. doi: 10.1146/annurev.biochem.68.1.89 10872445

[B45] Kuzyakov Y. Blagodatskaya E. (2015). Microbial hotspots and hot moments in soil: concept & review. Soil Biol. Biochem. 83, 184–199. doi: 10.1016/j.soilbio.2015.01.025

[B46] Laughlin D. C. Messier J. (2015). Fitness of multidimensional phenotypes in dynamic adaptive landscapes. Trends Ecol. Evol. 30, 487–496. doi: 10.1016/j.tree.2015.06.003 26122484

[B47] Li F. Peng Y. Natali S. M. Chen K. Han T. Yang G.. (2017a). Warming effects on permafrost ecosystem carbon fluxes associated with plant nutrients. Ecology 98, 2851–2859. doi: 10.1111/j.1600-0587.2010.06904.x 28766706

[B48] Li H. Crabbe M. Xu F. Wang W. Niu R. Gao X.. (2017b). Seasonal variations in carbon, nitrogen and phosphorus concentrations and C:N:P stoichiometry in the leaves of differently aged *Larix principis-rupprechtii* mayr. plantations. Forests 8, 373. doi: 10.3390/f8100373 PMC5609765 28938020

[B49] Li H. Liu B. McCormack M. L. Ma Z. Guo D. (2017c). Diverse belowground resource strategies underlie plant species coexistence and spatial distribution in three grasslands along a precipitation gradient. New Phytol. 216, 1140–1150. doi: 10.1111/nph.14710 28758691

[B50] Li M. Huang C. Yang T. Drosos M. Wang J. Kang X.. (2019). Role of plant species and soil phosphorus concentrations in determining phosphorus: Nutrient stoichiometry in leaves and fine roots. Plant Soil 445, 231–242. doi: 10.1007/s11104-019-04288-3

[B51] Li T. Sun J. Fu Z. (2021). Halophytes differ in their adaptation to soil environment in the yellow river delta: Effects of water source, soil depth, and nutrient stoichiometry. Front. Plant Sci. 12. doi: 10.3389/fpls.2021.675921 PMC8204056 34140965

[B52] Li T. Zhang Z. Sun J. Fu Z. Zhao Y. Xu W. (2022). Seasonal variation characteristics of c, n, and p stoichiometry and water use efficiency of *Messerschmidia sibirica* and its relationship with soil nutrients. Front. Ecol. Evol. 10. doi: 10.3389/fevo.2022.948682

[B53] Lin G. Zeng D.-H. Mao R. (2020). Traits and their plasticity determine responses of plant performance and community functional property to nitrogen enrichment in a boreal peatland. Plant Soil 449, 151–167. doi: 10.1007/s11104-020-04478-4

[B54] Liu F. Liu Y. Wang G. Song Y. Liu Q. Li D.. (2015). Seasonal variations of c: N: P stoichiometry and their trade-offs in different organs of *Suaeda salsa* in coastal wetland of yellow river delta, China. PloS One 10, e0138169. doi: 10.1371/journal.pone.0138169 26393356 PMC4578878

[B55] Liu J. Fang X. Tang X. Wang W. Zhou G. Xu S.. (2019). Patterns and controlling factors of plant nitrogen and phosphorus stoichiometry across china’s forests. Biogeochemistry 143, 191–205. doi: 10.1007/s10533-019-00556-7

[B56] Liu D. Zhang J. Biswas A. Cao J. Xie H. Qi X. (2020). Seasonal dynamics of leaf stoichiometry of *Phragmites australis*: a case study from yangguan wetland, dunhuang, China. Plants 9, 1323. doi: 10.3390/plants9101323 33036307 PMC7600640

[B57] Liu R. Wang D. (2021). C:N:P stoichiometric characteristics and seasonal dynamics of leaf-root-litter-soil in plantations on the loess plateau. Ecol. Indic. 127, 107772. doi: 10.1016/j.ecolind.2021.107772

[B58] Lloret F. Escudero A. Iriondo J. M. Martínez-Vilalta J. Valladares F. (2012). Extreme climatic events and vegetation: the role of stabilizing processes. Global Change Biol. 18, 797–805. doi: 10.1111/j.1365-2486.2011.02624.x

[B59] Luo W. Zuo X. Ma W. Xu C. Li A. Yu Q.. (2018). Differential responses of canopy nutrients to experimental drought along a natural aridity gradient. Ecology 99, 2230–2239. doi: 10.1002/ecy.2444 30157292

[B60] Luo Y. Luo Y. Peng Q. Li K. Gong Y. Han W. (2021). Patterns of nitrogen and phosphorus stoichiometry among leaf, stem and root of desert plants and responses to climate and soil factors in xinjiang, China. Catena 199, 105100. doi: 10.1016/j.catena.2020.105100

[B61] Loupassaki M. Chartzoulakis K. Digalaki N. Androulakis I. (2002). Effects of salt stress on concentration of nitrogen, phosphorus, potassium, calcium, magnesium, and sodium in leaves, shoots, and roots of six olive cultivars. J. Plant Nutr. 25, 2457–2482. doi: 10.1081/pln-120014707

[B62] Mayor J. R. Wright S. J. Turner S. J. (2014). Species-specific responses of foliar nutrients to longterm nitrogen and phosphorus additions in a lowland tropical forest. J. Ecol. 102, 36–44. doi: 10.1111/1365-2745.12190

[B63] Minden V. Kleyer M. (2014). Internal and external regulation of plant organ stoichiometry. Plant Biol. 16, 897–907. doi: 10.1111/plb.12155 24552639

[B64] Mouillot D. Bellwood D. R. Baraloto C. Chave J. Galzin R. Harmelin-Vivien M.. (2013). Rare species support vulnerable functions in high-diversity ecosystems. PLoS Biol. 11, e1001569. doi: 10.1371/journal.pbio.1001569 23723735 PMC3665844

[B65] Muscarella R. Uriarte M. (2016). Do community-weighted mean functional traits reflect optimal strategies? Proc. R. Soc B. 283, 20152434. doi: 10.1098/rspb.2015.2434 PMC4822452 27030412

[B66] National Soil Census Office of China (1992). Soil census techniques in China (Beijing: Agriculture Press).

[B67] Niklas K. J. Cobb E. C. (2005). N, p and c stoichiometry of *Eranthis hyemalis* (Ranunculaceae) and the allometry of plant growth. Am. J. Bot. 92, 1256–1263. doi: 10.2307/4126089 21646146

[B68] Parida A. K. Das A. Mittra B. (2004). Effects of salt on growth, ion accumulation, photosynthesis and leaf anatomy of the mangrove, *Bruguiera parviflora* . Trees 18, 167–174. doi: 10.1007/s00468-003-0293-8

[B69] Patel A. D. Jadeja H. Pandey A. N. (2010). Effect of salinization of soil on growth, water status and nutrient accumulation in seedlings of *Acacia auriculiformis* (Fabaceae). J. Plant Nutr. 33, 914–932. doi: 10.1080/01904161003669939

[B70] Pérez-Ramos I. M. Matías L. Gómez-Aparicio L. Godoy Ó. (2019). Functional traits and phenotypic plasticity modulate species coexistence across contrasting climatic conditions. Nat. Commun. 10, 2555. doi: 10.1038/s41467-019-10453-0 31186418 PMC6560116

[B71] Qiang Z. Liu Q. Yin H. Zhao C. Lin Z. Zhou G. (2018). C:N:P stoichiometry of ericaceae species in shrubland biomes across southern China: Influences of climate, soil and species identity. J. Plant Ecol. 2, 346–357. doi: 10.1093/jpe/rty033

[B72] Ramoliya P. J. Patel H. M. Joshi J. B. Pandey A. N. (2006). Effect of salinization of soil on growth and nutrient accumulation in seedlings of *Prosopis cineraria.* J. Plant Nutr. 29, 283–303. doi: 10.1080/01904160500476806

[B73] Reich P. B. Oleksyn J. (2004). Global patterns of plant leaf n and p in relation to temperature and latitude. Proc. Natl. Acad. Sci. U.S.A. 101, 11001–11006. doi: 10.1073/pnas.0403588101 15213326 PMC503733

[B74] Reich P. B. Tjoelker M. G. Pregitzer K. S. Wright I. J. Oleksyn J. Machado J. L. (2008). Scaling of respiration to nitrogen in leaves, stems and roots of higher land plants. Ecol. Lett. 793–801. doi: 10.1111/j.1461-0248.2008.01185.x 18445031

[B75] Reich P. B. Oleksyn J. Wright I. J. (2009). Leaf phosphorus influences the photosynthesis-nitrogen relation: a cross-biome analysis of 314 species. Oecologia 160, 207–212. doi: 10.2307/40310023 19212782

[B76] Ren S. J. Yu G. R. Tao B. Wang S. Q. (2007). Leaf nitrogen and phosphorus stoichiometry across 654 terrestrial plant species in NSTEC. J. Environ. Sci. China 28, 2665–2673. doi: 10.13227/j.hjkx.2007.12.007 18300391

[B77] Rong Q. Liu J. Cai Y. Lu Z. Zhao Z. Yue W.. (2015). Leaf carbon, nitrogen and phosphorus stoichiometry of *Tamarix chinensis* lour. in the laizhou bay coastal wetland, China. Ecol. Eng. 76, 57–65. doi: 10.1016/j.ecoleng.2014.03.002

[B78] Salazar-Tortosa D. Castro J. Villar-Salvador P. Viñegla B. Matías L. Michelsen A.. (2018). The “isohydric trap”: a proposed feedback between water shortage, stomatal regulation, and nutrient acquisition drives differential growth and survival of European pines under climatic dryness. Glob. Change Biol. 24, 4069–4083. doi: 10.1111/gcb.14311 29768696

[B79] Sardans J. Peñuelas J. (2008). Drought changes nutrient sources, content and stoichiometry in the bryophyte *Hypnum cupressiforme* hedw. growing in a Mediterranean forest. J. Bryol. 30, 59–65. doi: 10.1179/174328208x281987

[B80] Sardans J. Peñuelas J. (2012). The role of plants in the effects of global change on nutrient availability and stoichiometry in the plant-soil system. Plant Physiol. 160, 1741–1761. doi: 10.1104/pp.112.208785 23115250 PMC3510107

[B81] Sardans J. Peñuelas J. (2014). Climate and taxonomy underlie different elemental concentrations and stoichiometrics of forest species: the optimum ‘‘biogeochemocal niche’’. Plant Ecol. 215, 441–455. doi: 10.1007/s11258-014-0314-2 25983614 PMC4430814

[B82] Shi L. Li Q. Fu X. Kou L. Dai X. Wang H. (2021). Foliar, root and rhizospheric soil C:N:P stoichiometries of overstory and understory species in subtropical plantations. Catena 198, 105020. doi: 10.1016/j.catena.2020.105020

[B83] Song M. Chai Q. Li X. Yao X. Li C. Christensen M. J.. (2015). An asexual *Epichloë* endophyte modifies the nutrient stoichiometry of wild barley (*Hordeum brevisubulatum*) under salt stress. Plant Soil 387, 153–165. doi: 10.1007/s11104-014-2289-0

[B84] Sun X. Gao Y. Wang D. Chen J. Zhang F. Zhou J.. (2017). Stoichiometric variation of halophytes in response to changes in soil salinity. Plant Biol. 19, 360–367. doi: 10.1111/plb.12552 28135015

[B85] Tessier J. T. Raynal D. J. (2003). Use of nitrogen to phosphorus ratios in plant tissue as an indicator of nutrient limitation and nitrogen saturation. J. Appl. Ecol. 40, 523–534. doi: 10.2307/3506024

[B86] Thompson K. Parkinson J. A. Band S. R. Spencer R. E. (2010). A comparative study of leaf nutrient concentrations in a regional herbaceous flora. New Phytol. 136, 679–689. doi: 10.1046/j.1469-8137.1997.00787.x 33863101

[B87] Umaña M. N. Zhang C. Cao M. Lin L. Swenson N. G. (2015). Commonness, rarity, and intraspecific variation in traits and performance in tropical tree seedlings. Ecol. Lett. 18, 1329–1337. doi: 10.1111/ele.12527 26415689

[B88] Violle C. Enquist B. J. Mcgill B. J. Jiang L. Albert C. H. Hulshof C.. (2012). The return of the variance: Intraspecific variability in community ecology. Trends Ecol. Evol. 27, 244–252. doi: 10.1016/j.tree.2011.11.014 22244797

[B89] Vitousek P. M. Porder S. Houlton B. Z. Chadwick O. A. (2010). Terrestrial phosphorus limitation: Mechanisms, implications, and nitrogen–phosphorus interactions. Ecol. Appl. 20, 5–15. doi: 10.1890/08-0127.1 20349827

[B90] Vrede T. Dobberfuhl D. R. Kooijman S. A. L. M. Elser J. J. (2004). Fundamental connections among organism c: N: P stoichiometry, macromolecular composition, and growth. Ecology 85, 1217–1229. doi: 10.1890/02-0249

[B91] Wang L. Zhao G. Li M. Zhang M. Zhang L. Zhang X.. (2015). C:N:P stoichiometry and leaf traits of halophytes in an arid saline environment, Northwest China. PloS One 10, e0119935. doi: 10.1371/journal.pone.0119935 25798853 PMC4370893

[B92] Wang C. Cong J. H. Wang K. Q. Y. Cai W. J. Li Y. L. Fu S.. (2021). Research on china’s technology lists for addressing climate change. Chin. J. Popul. Resour. Environ. 19, 151–161. doi: 10.1016/j.cjpre.2021.12.017

[B93] Weih M. L. Bonosi L. Ghelardini L. Ronnberg-Wastljung A. C. (2011). Optimizing nitrogen economy under drought: Increased leaf nitrogen is an acclimation to water stress in willow (*Salix* spp.). Ann. Bot. 108, 1347–1353. doi: 10.1093/aob/mcr227 21896572 PMC3197455

[B94] Werner T. P. Amrhein N. Freimoser F. M. (2007). Inorganic polyphosphate occurs in the cell wall of *Chlamydomonas reinhardtii* and accumulates during cytokinesis. BMC Plant Biol. 7, 51. doi: 10.1186/1471-2229-7-51 17892566 PMC2096623

[B95] Wu T.-G. Chen B.-F. Xiao Y.-H. Pan Y.-J. Chen Y. Xiao J.-H. (2010). Leaf stoichiometry of trees in three forest types in pearl river delta, south china. chin. J. Plant Ecol. 34, 58–63. doi: 10.3773/j.issn.1005-264x.2010.01.009

[B96] Xiong X.-S. Cai H.-Y. Li Y.-Q. Ma W.-H. Niu K.-C. Chen D.-M.. (2020). Seasonal dynamics of leaf c, n and p stoichiometry in plants of typical steppe in nei Mongol, china. chin. J. Plant Ecol. 44, 1138–1153. doi: 10.17521/cjpe.2020.0105

[B97] Xiong J. Dong L. Lu J. Hu W. Gong H. Xie S.. (2022). Variation in plant carbon, nitrogen and phosphorus contents across the drylands of China. Funct. Ecol. 36, 174–186. doi: 10.1111/1365-2435.13937

[B98] Yan K. Duan C. Fu D. Li J. Wong M. H. G. Qian L.. (2015). Leaf nitrogen and phosphorus stoichiometry of plant communities in geochemically phosphorus-enriched soils in a subtropical mountainous region, SW China. Environ. Earth Sci. 74, 3867–3876. doi: 10.1007/s12665-015-4519-z

[B99] Yan Z. Li P. Chen Y. Han W. Fang J. (2016). Nutrient allocation strategies of woody plants: An approach from the scaling of nitrogen and phosphorus between twig stems and leaves. Sci. Rep. 6, 20099. doi: 10.1038/srep20099 26848020 PMC4742826

[B100] Yang X. Chi X. Ji C. Liu H. Ma W. Mohhammat A.. (2016). Variations of leaf n and p concentrations in shrubland biomes across northern China: Phylogeny, climate, and soil. Biogeosciences 13, 4429–4438. doi: 10.5194/bg-13-4429-2016

[B101] Yang Y. Liu B. An S. (2018). Ecological stoichiometry in leaves, roots, litters and soil among different plant communities in a desertified region of northern China. Catena 166, 328–338. doi: 10.1016/j.catena.2018.04.018

[B102] Zeng Q. Li X. Dong Y. Li Y. Cheng M. (2015). Ecological stoichiometry characteristics and physical-chemical properties of soils at different latitudes on the loess plateau. J. Nat. Resour. 30, 870–879. doi: 10.11849/zrzyxb.2015.05.014

[B103] Zhang J. Zhao N. Liu C. Yang H. Li M. Yu G.. (2018). C:N:P stoichiometry in china’s forests: From organs to ecosystems. Funct. Ecol. 32, 50–60. doi: 10.1111/1365-2435.12979

[B104] Zhang A. Li X. Wu S. Li L. Jiang Y. Wang R.. (2021a). Spatial pattern of C:N:P stoichiometry characteristics of alpine grassland in the altunshan nature reserve at north qinghai-Tibet plateau. Catena 207, 105691. doi: 10.1016/j.catena.2021.105691

[B105] Zhang J. Li M. Xu L. Zhu J. Dai G. He N. (2021b). C:N:P stoichiometry in terrestrial ecosystems in China. Sci. Total Environ. 795, 148849. doi: 10.1016/j.scitotenv.2021.148849 34246133

[B106] Zhang J. Xie H. Biswas A. Shan Y. Qi X. Cao J. (2021c). Response of different organs’ stoichiometry of *Phragmites australis* to soil salinity in arid marshes, China. Glob. Ecol. Conserv. 31, e01843. doi: 10.1016/j.gecco.2021.e01843

[B107] Zhou X. Q. Guo Z. Y. Chen C. R. Jia Z. J. (2017). Soil microbial community structure and diversity are largely influenced by soil pH and nutrient quality in 78-year-old tree plantations. Biogeosciences 14, 2101–2111. doi: 10.5194/bg-14-2101-2017

[B108] Zhou Y. Jiao L. Qin H. Li F. (2021). Effect of environmental stress on the nutrient stoichiometry of the clonal plant *Phragmites australis* in inland riparian wetlands of Northwest China. Front. Plant Sci. 12. doi: 10.3389/fpls.2021.705319 PMC8416684 34490007

