# Supplementary material for: C:N:P stoichiometric variations of herbs and its relationships with soil properties and species relative abundance along the Xiaokai River irrigation in the Yellow River Delta, China
Source: Front Plant Sci. 2023 Jan 30;14:1130477. doi: 10.3389/fpls.2023.1130477 (PMC9923178; doi:10.3389/fpls.2023.1130477)
Supplement: Supplementary file 1 [file DataSheet_1.docx]

Supplementary Material

**Supplementary Table 1** The family and sampling number for plants’ C, N, P stoichiometries measurement of studied species

| Species | Family | N_Early_ | N_Late_ | N_Pooling_ |
| --- | --- | --- | --- | --- |
| *Setaria viridis* | Gramineae | 17 | 22 | 39 |
| *Phragmites australis* | Gramineae | 13 | 12 | 25 |
| *Artemisia scoparia* | Asteraceae | 15 | 10 | 25 |
| *Suaeda glauca* | Chenopodiaceae | 13 | 10 | 23 |
| *Chenopodium ficifolium* | Chenopodiaceae | 12 | 5 | 17 |
| *Humulus scandens* | [Moraceae](http://www.iplant.cn/info/Moraceae?t=z) | 6 | 6 | 12 |
| *Salsola collina* | Chenopodiaceae | 4 | 8 | 12 |
| *Metaplexis japonica* | Asclepiadaceae | 8 | / | 8 |
| *Cirsium arvense* | Asteraceae | 4 | / | 4 |
| *Digitaria sanguinalis* | Gramineae | / | 13 | 13 |
| *Chloris virgata* | Gramineae | / | 13 | 13 |
| *Sonchus oleraceus* | Asteraceae | / | 7 | 7 |
| *Cynanchum chinense* | Asclepiadaceae | / | 7 | 7 |
| *Gueldenstaedtia verna* | Fabaceae | / | 3 | 3 |
| *Rorippa indica* | [Cruciferae](http://www.iplant.cn/info/Cruciferae?t=z) | / | 3 | 3 |
| *Chenopodium album* | Chenopodiaceae | / | 3 | 3 |
| *Atriplex patens* | Chenopodiaceae | / | / | 3 |
| *Xanthium strumarium* | Asteraceae | / | / | 3 |

N_Early_, sampling number in the early growth stage; N_Late_, sampling number in the late growth stage; N_Pooling_, sampling number pooling the early and late growth stages.

**Supplementary Table 2** Relationships between the above- and below-ground parts C, N, P stoichiometries pooling the early and late growth stages

|  | AC | AN | AP | AC:N | AC:P | AN:P |
| --- | --- | --- | --- | --- | --- | --- |
| BC | -0.109 | 0.031 | 0.022 | -0.124 | -0.159 | -0.128 |
| BN | **-0.359** | **0.562** | -0.189 | **-0.507** | 0.192 | **0.613** |
| BP | **-0.444** | **0.354** | 0.123 | **-0.400** | *-0.238* | 0.085 |
| BC:N | **0.418** | **-0.518** | 0.101 | **0.502** | -0.068 | **-0.483** |
| BC:P | **0.380** | **-0.333** | -0.139 | **0.332** | 0.229 | -0.099 |
| BN:P | -0.082 | **0.378** | **-0.324** | **-0.311** | **0.405** | **0.647** |

Fond in bold when *P* < 0.05 and in italic when *P* < 0.1. AC, aboveground part C concentration; AN, aboveground part N concentration; AP, aboveground part P concentration; AC:N, aboveground part C:N ratio; AC:P, aboveground part C:P ratio; AN:P, aboveground part N:P ratio; BC, belowground part C concentration; BN, belowground part N concentration; BP, belowground part P concentration; BC:N, belowground part C:N ratio; BC:P, belowground part C:P ratio; BN:P, belowground part N:P ratio.

**Supplementary Table 3** Coefficient of variation (%) in plant C, N, P stoichiometries along the Xiaokai River irrigation

| Parameter | The early growth stage | The late growth stage | Pooling the two growth stages |
| --- | --- | --- | --- |
| AC | 5.757 | 2.261 | 5.140 |
| AN | 23.534 | 30.853 | 32.880 |
| AP | 8.189 | 39.912 | 29.219 |
| AC:N | 22.699 | 36.409 | 41.589 |
| AC:P | 10.445 | 57.455 | 46.430 |
| AN:P | 26.434 | 61.428 | 44.778 |
| BC | 4.712 | 4.797 | 4.951 |
| BN | 51.190 | 35.909 | 43.465 |
| BP | 34.424 | 24.407 | 30.466 |
| BC:N | 35.581 | 42.484 | 39.099 |
| BC:P | 34.875 | 22.626 | 32.653 |
| BN:P | 38.467 | 31.574 | 35.202 |

AC, aboveground part C concentration; AN, aboveground part N concentration; AP, aboveground part P concentration; AC:N, aboveground part C:N ratio; AC:P, aboveground part C:P ratio; AN:P, aboveground part N:P ratio; BC, belowground part C concentration; BN, belowground part N concentration; BP, belowground part P concentration; BC:N, belowground part C:N ratio; BC:P, belowground part C:P ratio; BN:P, belowground part N:P ratio.


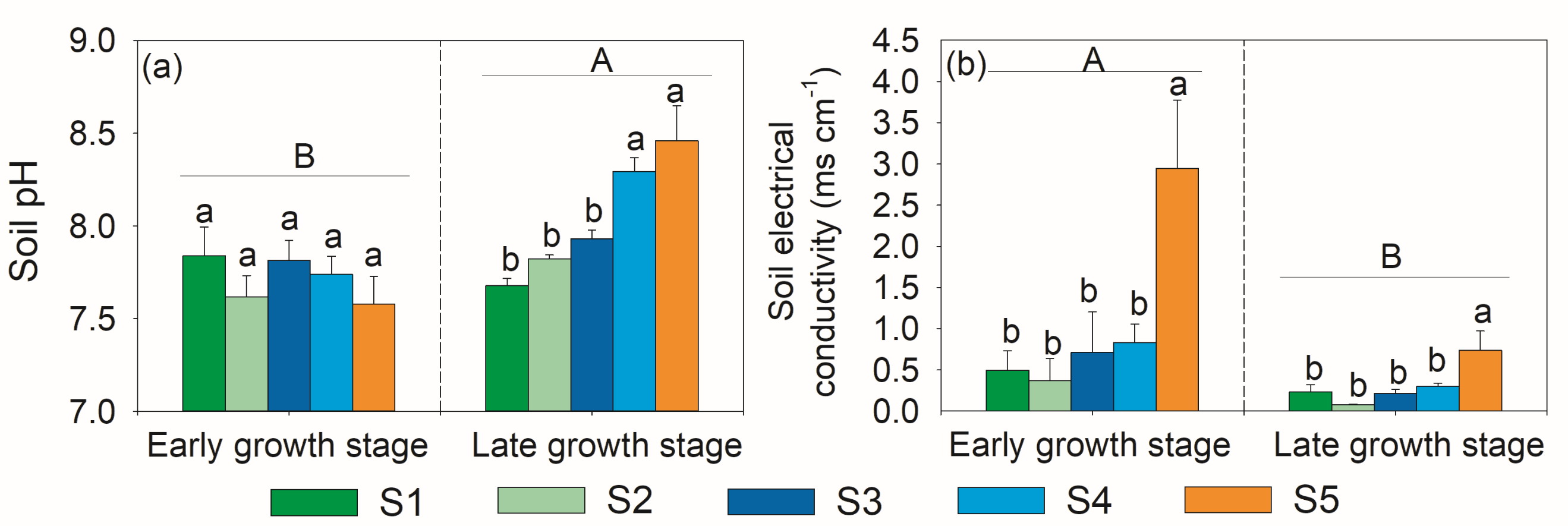


**Supplementary Figure 1** Variation patterns of soil pH and electrical conductivity in subsoil layer. Different lowercase letters indicate significant differences amongst plots. Different uppercase letters indicate significant differences between sampling seasons.


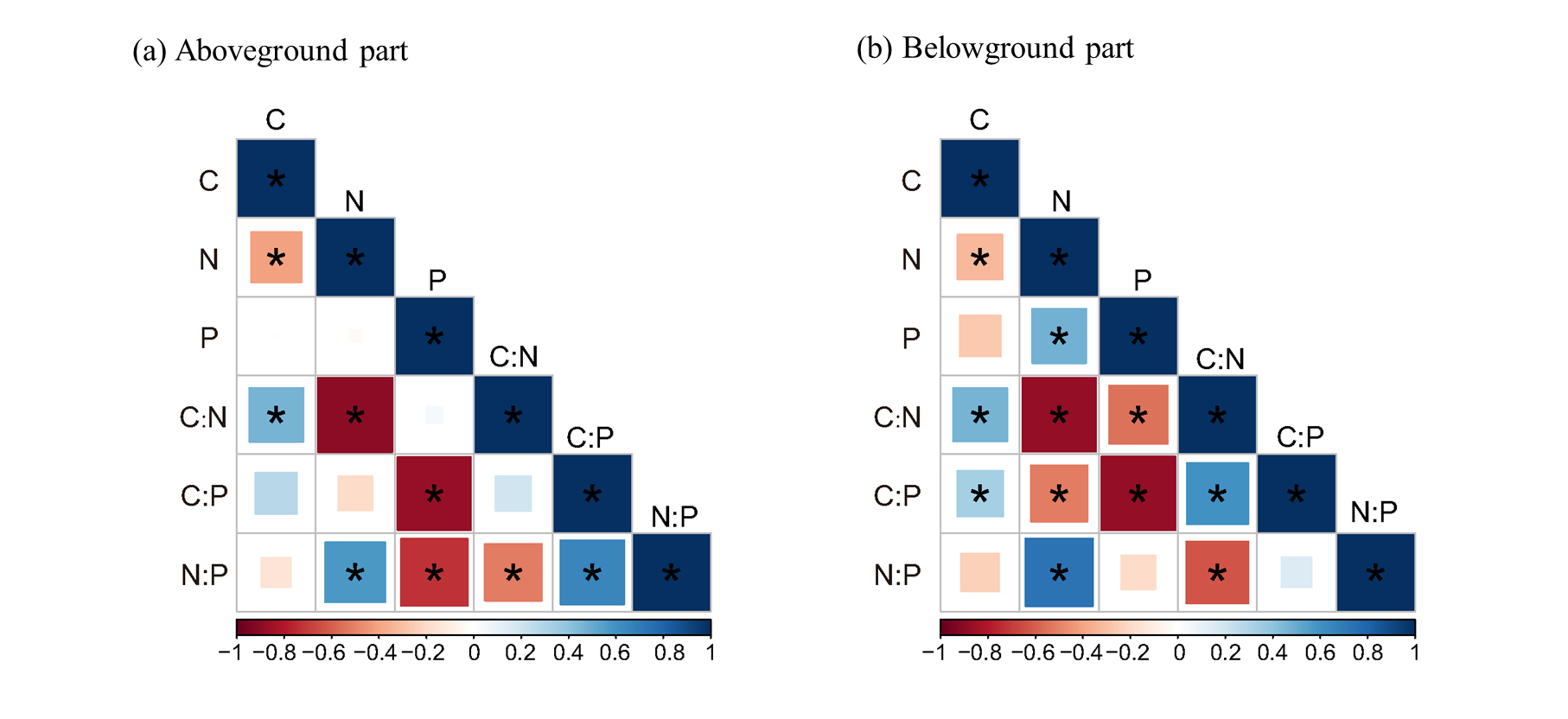


**Supplementary Figure 2** Relationships between the above- and belowground parts C:N:P stoichiometries pooling the two growth stages.


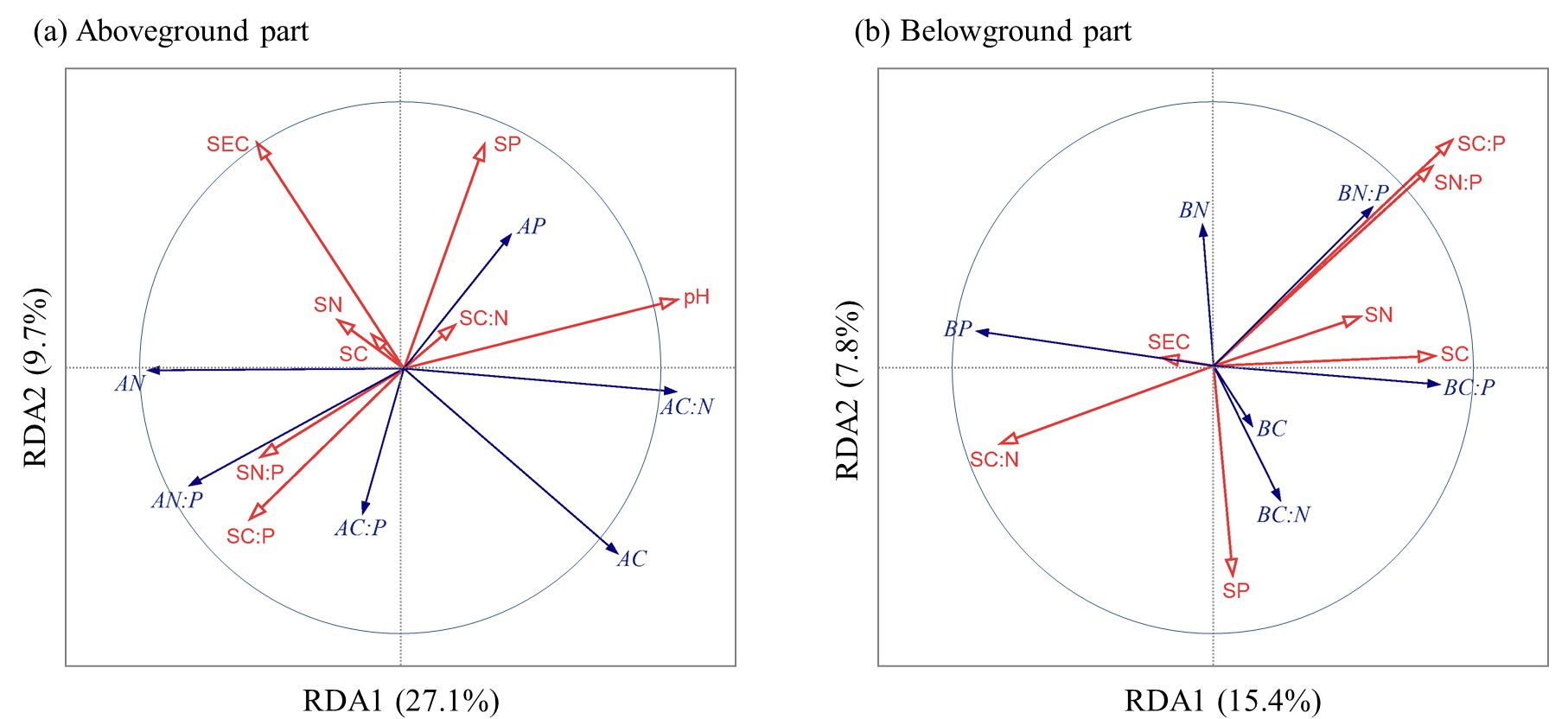


**Supplementary Figure 3** RDA-ordination biplot of the above- and belowground parts C:N:P stoichiometries and soil properties pooling the two growth stages. AC, aboveground part C concentration; AN, aboveground part N concentration; AP, aboveground part P concentration; AC:N, aboveground part C:N ratio; AC:P, aboveground part C:P ratio; AN:P, aboveground part N:P ratio; BC, belowground part C concentration; BN, belowground part N concentration; BP, belowground part P concentration; BC:N, belowground part C:N ratio; BC:P, belowground part C:P ratio; BN:P, belowground part N:P ratio; SC, soil C concentration; SN, soil N concentration; SP, soil P concentration; SC:N, soil C:N ratio; SC:P, soil C:P ratio; SN:P, soil N:P ratio; pH, soil pH; SEC, soil electrical conductivity.


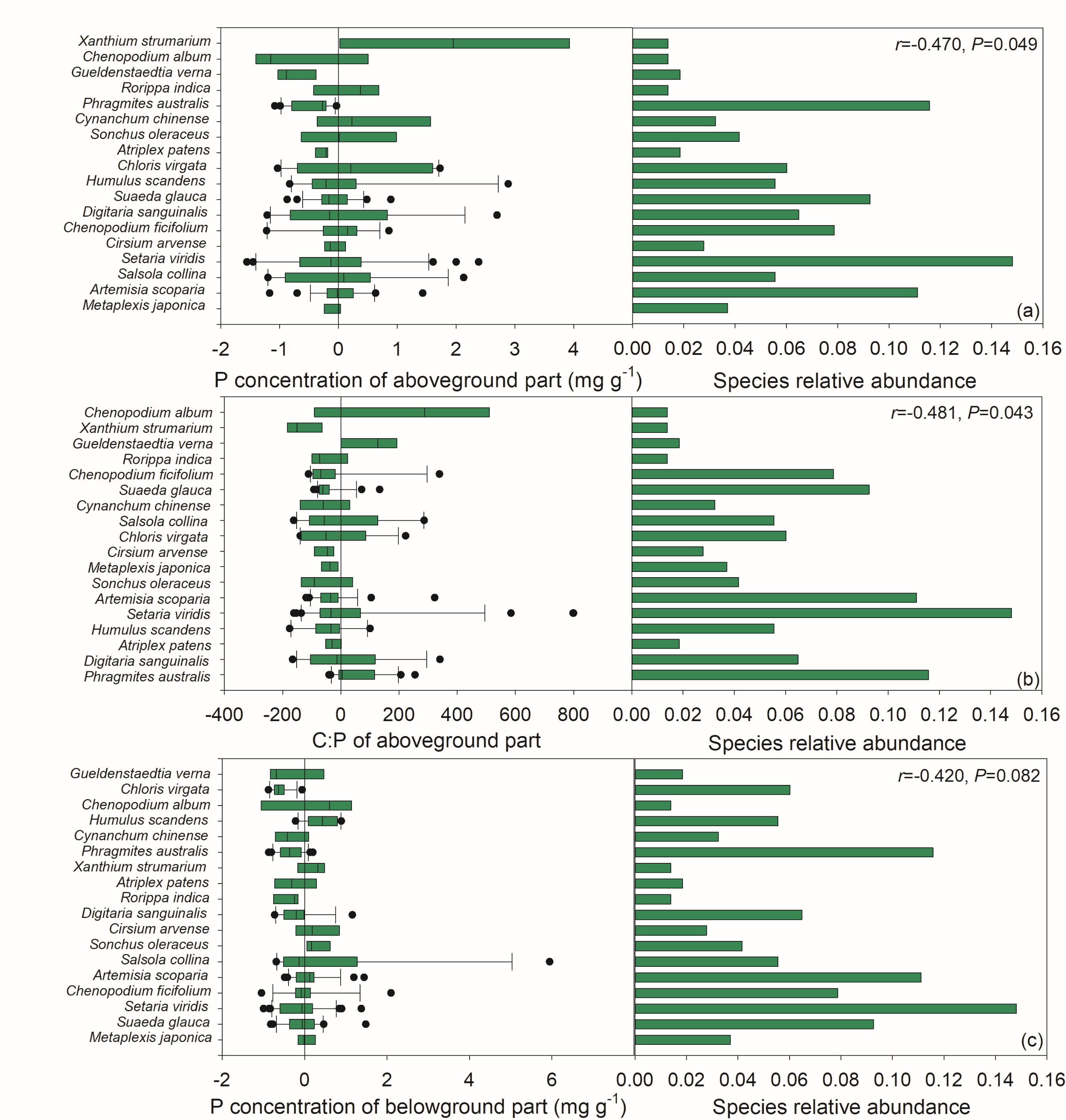


**Supplementary Figure 4** The relative position of species C:N:P stoichiometries distribution across sampling sites upon pooling the two growth stages. Left panel: the x-axis represents the difference between the median C, N, P stoichiometries for each species and the community-weighted mean (CWM) C, N, P stoichiometries for the entire plant community. The y-axis arrays species from bottom to top based on their distance how close to the CWM value. Each boxplot represents the distribution of plants’ C, N, P stoichiometries of each species. Right panel: species relative abundance across sampling sites. The *r*-value of Pearson correlation analysis of the absolute values of the differences between the median C, N, P stoichiometries of each species and the CWM value of the entire community against their species relative abundance is provided at the upper right.


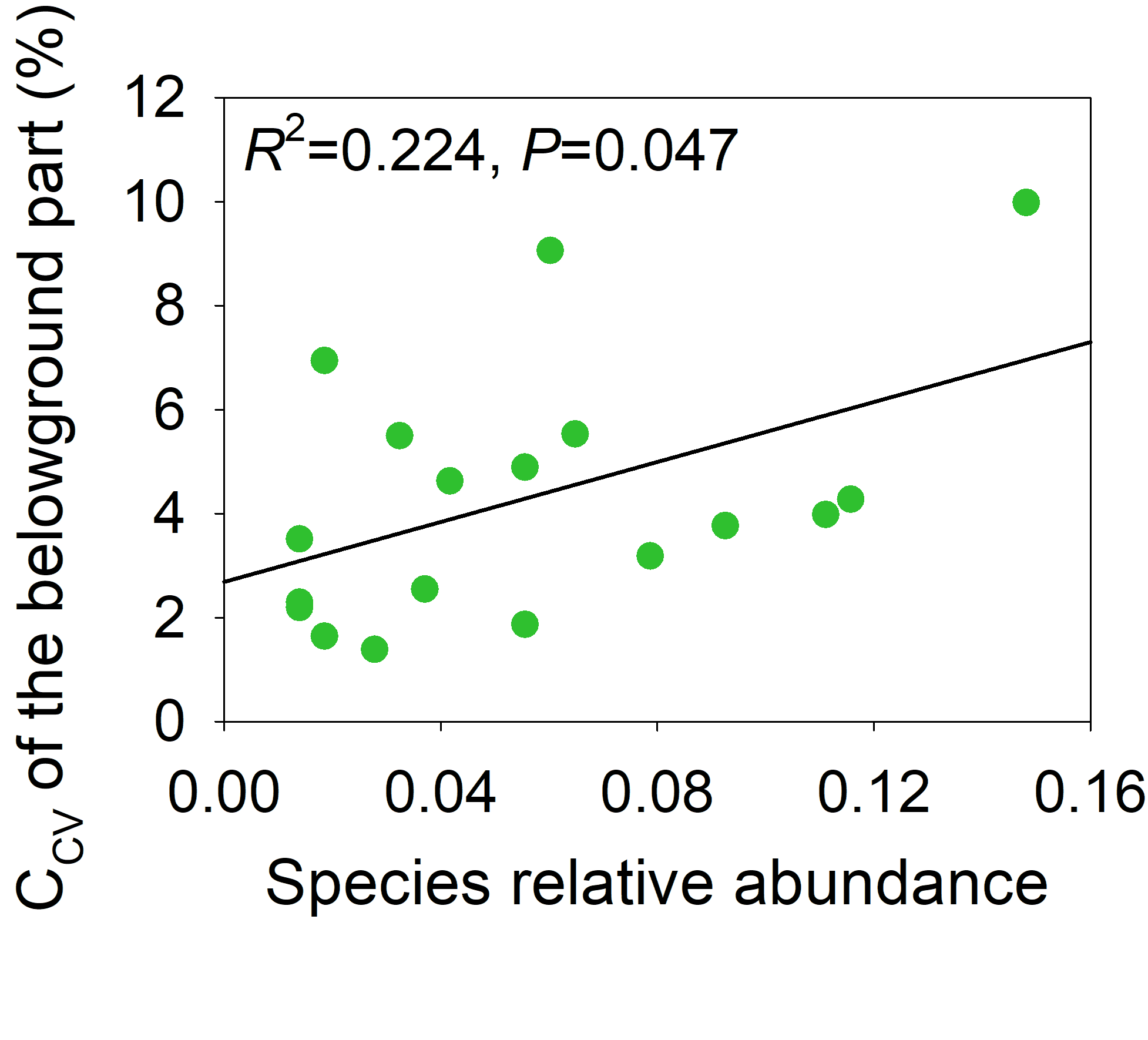


**Supplementary Figure 5** Relationship between intraspecific variation of C concentration (C_CV_) of the belowground part in relation to species relative abundance upon pooling the two growth stages.
